# Acute‐Phase Interventions After Self‐Harm for Preventing Suicide and Recurrence: A Systematic Review and Meta‐Analysis

**DOI:** 10.1111/acps.70085

**Published:** 2026-03-09

**Authors:** Guilherme Pimenta Roncete, Luis C. Farhat, Loren Beiram, Bianca Besteti Fernandes Damiano, Craig J. Bryan, Karina L. Ramirez, Euripedes Constantino Miguel, Rodolfo Furlan Damiano

**Affiliations:** ^1^ Departamento e Instituto de Psiquiatria Hospital das Clínicas, Faculdade de Medicina, Universidade de São Paulo São Paulo Brazil; ^2^ Program for Education, Research, and Care in Treatment‐Resistant Depression, Self‐Injury, and Suicidality, Hospital das Clínicas, Faculdade de Medicina Universidade de São Paulo São Paulo Brazil; ^3^ University of Vermont Burlington Vermont USA; ^4^ Child Study Center Yale School of Medicine New Haven Connecticut USA

**Keywords:** brief intervention, meta‐analysis, self‐harm, suicide prevention

## Abstract

**Introduction:**

Suicide risk is markedly elevated following presentation after self‐harm, yet the evidence base for interventions initiated in the immediate post‐presentation period has not been synthesized with specific attention to this high‐risk window. Clarifying which early interventions may reduce adverse outcomes is essential for informing psychiatric care and prevention strategies.

**Methods:**

We conducted a systematic review and meta‐analysis of randomized clinical trials enrolling individuals presenting to clinical services after self‐harm, with interventions initiated within 1 month of the index episode. PubMed, Embase, PsycINFO, the WHO International Clinical Trials Registry Platform (ICTRP), and ClinicalTrials.gov were searched from inception through April 24, 2025. Two reviewers independently screened studies and extracted data using standardized forms. Risk of bias was assessed using the Cochrane RoB 2 tool, and certainty of evidence was evaluated with GRADE. Random‐effects meta‐analyses with Hartung–Knapp adjustment were conducted for intervention categories with at least three trials, and prediction intervals were calculated. Prespecified meta‐regressions examined participant‐ and intervention‐level moderators.

**Results:**

Sixty randomized clinical trials, including 22,654 participants, met the inclusion criteria. Across pooled analyses, no intervention category was associated with a statistically significant reduction in repeat self‐harm at any follow‐up interval. For suicide deaths, problem‐solving therapy was associated with lower mortality at medium‐term follow‐up (6–12 months; OR 0.45, 95% CI 0.29–0.70; *I* ^2^ = 0%), and brief intervention and contact was associated with lower mortality at long‐term follow‐up (> 12 months; OR 0.34, 95% CI 0.15–0.79; *I* ^2^ = 0%). Meta‐regression analyses indicated that younger mean sample age and trial inclusion based on suicidal intent moderated effects on repeat self‐harm at > 6–12 months.

**Conclusions:**

Among trials initiating treatment within 1 month of self‐harm presentation, effects on repeat self‐harm were small and not statistically significant, likely reflecting outcome heterogeneity in the acute post‐presentation period. In contrast, structured problem‐solving and brief contact‐based interventions were associated with lower suicide mortality at medium‐ and long‐term follow‐up. These findings support the use of scalable early interventions in psychiatric services and highlight priorities for future trials powered to detect effects on suicide mortality.

**Trial Registration:** PROSPERO: CRD42023458233

## Key Points

**Summations:**

○ Interventions initiated within 1 month after a self‐harm presentation showed limited effects on repeat self‐harm, underscoring the challenges of this outcome in acute post‐presentation contexts.
○ Problem‐solving therapy and brief intervention and contact were associated with lower suicide mortality at medium‐ and long‐term follow‐up, highlighting the potential value of scalable early strategies.
○ Focusing on time‐restricted high‐risk periods offers a clinically relevant framework for evaluating preventive interventions in psychiatric services.

**Limitations:**

○ Suicide deaths were rare events across trials, limiting statistical power and contributing to uncertainty despite consistent directional effects.
○ Considerable heterogeneity in definitions, measurement, and follow‐up of repeat self‐harm may have reduced the ability to detect intervention effects on this outcome.
○ Many trials were not designed specifically for the acute post‐presentation period, constraining inference about optimal timing and sequencing of interventions.

## 1 Introduction

Self‐harm and suicide attempts constitute a global public health crisis of unprecedented magnitude, with profound consequences for individuals, families, and healthcare systems worldwide. The relationship between self‐harm and subsequent suicide is particularly alarming: individuals who engage in self‐harm face a 16‐fold increased risk of dying by suicide compared to the general population [1]. This risk peaks immediately after the self‐harm episode. For instance, previous evidence indicated suicide rates of approximately 439 per 100,000 person‐years, about 37 times higher than the general population, in the year following self‐harm, and the risk of suicide in the first month is approximately 10 times higher than during the subsequent 11 months among those who survive self‐harm [2]. The acute period of risk following self‐harm, consistently highlighted in cumulative incidence estimates and Kaplan–Meier survival analyses from large cohort studies, remains systematically underrepresented in intervention research, marking a critical target for suicide prevention efforts [3, 4].

The evidence base for interventions following self‐harm has expanded substantially over recent decades. Clinical services have become key settings for initiating preventive strategies following self‐harm presentation, with studies showing that psychosocial interventions, such as active contact and follow‐up interventions and brief intervention and contact (BIC), can significantly reduce the frequency of suicide re‐attempts or deaths by suicide within 1–2 years after the index attempt [5]. Additionally, specific therapeutic approaches, such as cognitive behavioral therapy (CBT), have emerged as potentially effective, with recent controlled trials showing substantial reductions in suicide attempts [6, 7, 8]. Similarly, dialectical behavior therapy (DBT), particularly its adolescent adaptation (DBT‐A), has demonstrated high‐certainty evidence for reducing self‐harm repetition [9]. Regarding pharmacological interventions, although psychosocial approaches dominate the evidence base, recent systematic reviews suggest emerging but limited evidence for certain medications. Specifically, antipsychotics and lithium have demonstrated potential benefits compared to placebo, while ketamine and esketamine seem to be promising. However, the overall certainty of evidence remains low to very low, emphasizing the need for more robust trials in this area [10, 11, 12].

Despite these advances, a sobering meta‐analysis by Fox et al. examining nearly 50 years of research found that overall intervention effects remain disappointingly small, with no substantial improvements in efficacy over time, indicating fundamental limitations in our current approaches [13]. Due to the challenges inherent in real‐world settings and patient heterogeneity, as well as the low incidence of outcomes, many trials and subsequent meta‐analyses have adopted varied inclusion criteria with heterogeneous recruitment timeframes, often enrolling patients up to 6 months or longer after their initial self‐harm episode, thereby diluting intervention effects relevant to the immediate post‐presentation phase [14, 15, 16]. Additionally, previous reviews have examined psychosocial and pharmacological interventions separately, preventing a comprehensive comparison across all treatment modalities [10, 16]. Moreover, there has been insufficient focus on evaluating effect modifiers such as psychiatric comorbidity, intervention intensity, age, sex, and history of repeated self‐harm. This gap restricts our ability to identify which interventions are most effective for specific subgroups and at particular time points, despite recommendations from the National Institute for Health and Care Excellence (NICE) guidelines to tailor interventions based on individual patient profiles [17].

A central feature of this review is the restriction to studies enrolling patients within 1 month of an index self‐harm episode resulting in healthcare presentation. Population‐based evidence consistently shows that suicide risk is highly concentrated early after self‐harm: U.S. data indicate the risk of suicide in the first month is approximately 10 times higher than during the subsequent 11 months [2], while French national data show that nearly one‐third of recurrences occur within this window [18]. Our methodological framework deliberately aligns with that of Inagaki et al., who similarly restricted inclusion to the first month [19, 20], rather than the broader 6‐month window adopted by the Cochrane reviews [9, 10, 16]. Given that Inagaki's most recent synthesis was published over 6 years ago, an updated review incorporating newly available trials is warranted.

This review focused on individuals with behaviorally verified self‐harm who presented to healthcare services, excluding ideation‐only samples due to their lower short‐term mortality risk and the methodological heterogeneity they introduce [21, 22]. Unlike prior syntheses that examined psychosocial and pharmacological interventions separately, we consider both modalities within a single time window, reflecting real‐world post‐presentation clinical decision‐making, in which different options are considered concurrently. We also examine potential effect modifiers, including psychiatric comorbidity, suicidal intent, intervention intensity, age, and history of repeated self‐harm. Our primary objective was to determine the comparative effectiveness of interventions in reducing repeated self‐harm and suicide deaths across multiple follow‐up periods (≤ 6 months, > 6–12 months, and > 12 months), with secondary objectives focused on identifying intervention characteristics that influence treatment effects. Both outcomes were prespecified as co‐primary; however, suicide deaths were emphasized in interpretation as the most clinically consequential endpoint in the post‐presentation period.

## 2 Aims of the Study

This systematic review and meta‐analysis aimed to determine the comparative effectiveness of pharmacological and non‐pharmacological interventions initiated within 1 month of a self‐harm presentation in reducing repeat self‐harm and suicide deaths across short‐, medium‐, and long‐term follow‐up periods. We also sought to identify participant‐level and intervention‐level characteristics that moderate treatment effects, including age, suicidal intent, and history of repeated self‐harm.

## 3 Methods

This systematic review and meta‐analysis was reported in accordance with the Preferred Reporting Items for Systematic Reviews and Meta‐Analyses (PRISMA) 2020 statement [23], with the completed checklist available in Supporting Information S1. The protocol was prospectively registered with PROSPERO (CRD42023458233) on 7 September 2023.

### 3.1 Definitions

We define self‐harm as any intentional act of self‐injury or self‐poisoning, regardless of suicidal intent, resulting in presentation to healthcare services (including emergency, inpatient, or outpatient mental health settings). The definition excludes minor self‐injury episodes that don't result in clinical presentation, focusing specifically on high‐risk cases relevant to acute healthcare settings. This definition aligns with common practice in clinical trials and international guidelines [17, 24, 25, 26].

For suicidal intent, we classified trials according to the inclusion criteria specified in each article's methods or protocol. Studies were considered “with suicidal intent” if the inclusion criteria explicitly required “self‐harm with suicidal intent” (or a similar term), or if terms such as “self‐harm” or “suicide attempt” were used in the methods and were clearly defined elsewhere in the article (e.g., in the introduction) as acts involving suicidal intent. In contrast, studies were classified as “regardless of suicidal intent” if the inclusion criteria did not distinguish between non‐suicidal self‐injury and self‐harm with suicidal intent, or if suicidal intent was not explicitly required.

### 3.2 Search Strategy and Inclusion/Exclusion Criteria

Searches were conducted in five databases: PubMed, Embase, PsycINFO, the WHO International Clinical Trials Registry Platform (ICTRP), and ClinicalTrials.gov, covering all records from database inception to 24 April 2025. The strategy combined Medical Subject Headings (MeSH) and free‐text terms related to self‐harm, suicide, prevention, treatment, and emergency care, and was tailored to identify randomized and controlled clinical trials. No restrictions were applied for language, publication status, or year of publication, and the complete Boolean strategies for each database are available in Supporting Information S2.

Studies were eligible for inclusion if they were RCTs comparing pharmacological or non‐pharmacological interventions for individuals who had engaged in self‐harm, with intervention initiation required to occur within 1 month of the index episode. Our review included both blinded and non‐blinded trials, as well as parallel or crossover RCTs. Eligible studies were restricted to populations that had presented to, or were referred to, clinical services following a self‐harm episode. Trials based exclusively on community, school‐based, or screening samples without clinical presentation were not eligible. Studies were also excluded if they only enrolled participants who had suicidal ideation without any self‐harm behavior, or whose most recent self‐harm episode had occurred more than 1 month before baseline assessment. Finally, we excluded studies that failed to report the outcome of interest in an available indexed publication or trial registry.

### 3.3 Study Selection

All references identified through the database searches were independently screened by two reviewers (GPR and LB) to assess potential eligibility. Any disagreements were resolved through discussion, with consultation of a third reviewer when consensus could not be reached. Then, potentially eligible studies were assessed in a detailed manner for inclusion using the same independent two‐reviewer procedure. When eligibility criteria were unclear or additional information was required, we contacted the corresponding authors for clarification. Additionally, reference lists of all included studies and relevant systematic reviews were manually searched to identify any additional eligible trials.

### 3.4 Data Extraction

Two reviewers (G.P.R. and K.L.R.) independently extracted data from all included studies using a standardized data extraction spreadsheet. Any discrepancies were resolved through discussion or, when necessary, with the involvement of a third reviewer.

For each trial, we extracted study (author names, title, DOI, year of publication, recruitment period, country where the study was conducted, funding sources, declared conflicts of interest, trial registration identifiers, masking procedures, number of participating centers, recruitment setting), participant (mean age with standard deviation, sex distribution, marital status, race/ethnicity composition, proportion of individuals with a lifetime history of self‐harm, proportion of individuals with major depressive disorder and borderline personality disorder, requirement of suicidal intent at the time of the index self‐harm episode), and treatment (delivery format, dose, duration, qualifications of intervention providers, fidelity monitoring procedures, description of control conditions, and intervention type) characteristics. Additional details on the categories within those characteristics are described in Supporting Information S3. Interventions were categorized according to the framework by two coauthors (R.F.D. and L.B.), a psychologist and a psychiatrist with clinical experience in suicidality studies and clinical practice.

### 3.5 Outcome Measures

Intention‐to‐treat data were prioritized whenever available; we extracted data through completer when intention‐to‐treat analyses were not reported, with careful recording of the number of participants assessed for each outcome at each time point.

Outcomes were assessed at four predefined time points relative to the treatment period after baseline (T0): short‐term (T1, ≤ 6 months), medium‐term (T2, > 6 to 12 months), and long‐term (T3, > 12 months). When multiple observations existed within a single time frame, we extracted data from the latest assessment within that period.

The co‐primary outcomes were the recurrence of self‐harm and suicide deaths. Recurrence of self‐harm was identified through multiple sources, including self‐report questionnaires, collateral reports from family or clinicians, clinical record reviews, or systematic research monitoring systems. When both clinical records and self‐report data were available for the same outcome, we prioritized clinical records due to their greater objectivity. At each time point, we recorded the number of participants who engaged in one or more new episodes of self‐harm, regardless of suicidal intent, occurring after or during the intervention period. Consistent with prior research in this field, within each follow‐up unit of analysis in our study (T1, T2, T3), we prioritized the longest‐term assessment of self‐harm recurrence available in each study, given that previous meta‐analyses have shown limited sustained effects at extended follow‐up periods [14, 16, 19]. Suicide deaths were verified through mortality registers or official records when available. We recorded the number of events at each time point and calculated appropriate effect sizes for meta‐analysis.

### 3.6 Risk of Bias Assessment

The risk of bias was independently assessed by two reviewers using the revised Cochrane Risk of Bias tool (RoB 2) [27], which provides a comprehensive framework for evaluating potential sources of bias in RCTs. The tool assesses five distinct domains: randomization process, deviations from intended interventions, missing outcome data, measurement of the outcome, and selection of the reported result, including evidence of selective outcome reporting. Each domain was rated as “low,” “some concerns,” or “high” risk of bias, with the overall judgment synthesized for each study‐outcome combination.

### 3.7 Data Analysis

Statistical analyses were conducted using R software (R Core Team, 2024; R Foundation for Statistical Computing, Vienna, Austria), primarily utilizing the meta [28] and metafor [29] packages for different aspects of the analyses.

We calculated odds ratios (ORs) with 95% confidence intervals (CIs) for dichotomous outcomes and pooled data with the generic inverse of the variance random‐effects meta‐analysis.

Continuity corrections of 0.5 were applied when zero events occurred in either the intervention or control arms (single‐zero studies) or in both arms (double‐zero studies) to enable the inclusion of all RCTs in the analyses. Because of the ongoing debate about whether to include double‐zero studies in meta‐analyses, we adopted two approaches: the primary analysis, including single‐zero but excluding double‐zero studies, and a secondary analysis also including them [30]. All analyses compared psychosocial interventions to TAU, using random‐effects models with Hartung‐Knapp adjustments to provide more conservative confidence intervals that better account for uncertainty in the between‐study variance estimate. Prediction intervals were calculated to reflect the expected range of true effects in future studies conducted in similar settings, providing a clinically interpretable measure of heterogeneity [31].

All eligible RCTs were included in the quantitative synthesis irrespective of individual risk of bias, in order to provide a comprehensive overview of the available evidence. Risk of bias assessments and certainty of evidence ratings were used to inform the interpretation and strength of conclusions rather than to determine study inclusion. Certainty of evidence was systematically evaluated using the GRADE framework, considering risk of bias, inconsistency, indirectness, imprecision, and publication bias, and managed in GRADEpro GDT software [32, 33]. Evidence derived from studies at high risk of bias (RoB 2) was explicitly downgraded, thereby informing the robustness of conclusions.

Subgroup analyses were conducted by intervention type but were restricted to categories with at least three studies to ensure sufficient statistical power and to enable a more reliable assessment of heterogeneity and inconsistency. This criterion explains why the total number of trials identified in the systematic review exceeds the number included in specific quantitative meta‐analyses and forest plots. Subgroups included BIC, CBT, Problem‐Solving Therapy (PST), Psychodynamic, Case Management, Supportive Therapy, Family Intervention, Selective Serotonin Reuptake Inhibitors (SSRIs), Safety Planning, Therapeutic Assessment, Help Sheet, General Practitioner (GP) Guideline, Omega‐3, and Typical Antipsychotic.

We conducted meta‐regression to explore the potential moderating effects of participant and study‐level characteristics on treatment outcomes. Variables examined included suicidal intent at the index episode (required vs. not required for inclusion), history of self‐harm (first episode vs. repeat self‐harm), proportion of major depressive disorder in the sample, marital status distribution, mean age, proportion of females, mean number of intervention sessions delivered, and year of participants' recruitment beginning. For moderators with significant results at a given time‐point, we performed subgroup forest plots stratified by that variable.

Small‐study effects and potential publication bias were evaluated through visual inspection of trim‐and‐fill funnel plots and statistical assessment of funnel plot asymmetry using Egger's test, performed for comparisons including 10 or more studies.

## 4 Results

### 4.1 Selection and Inclusion of Studies

**FIGURE 1:**
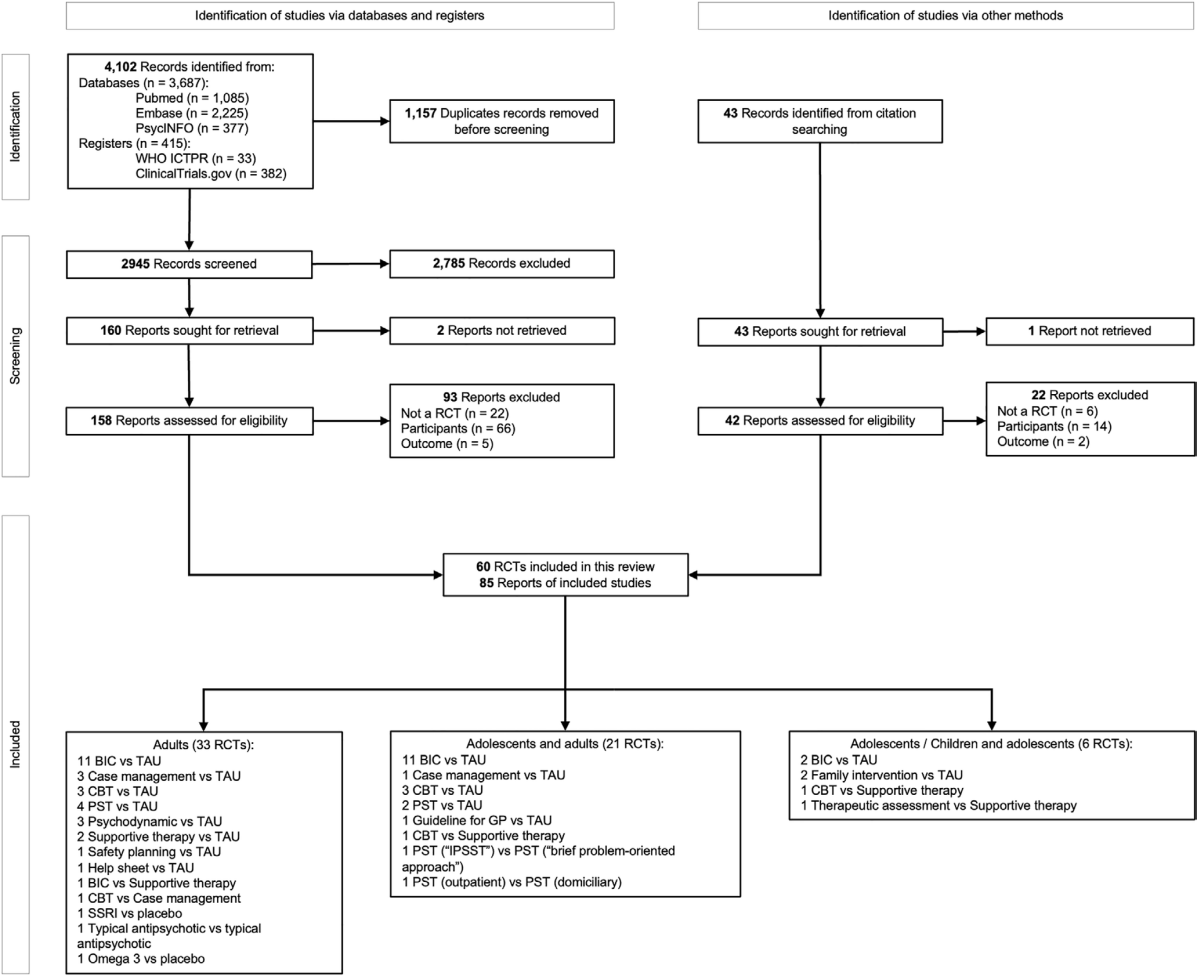
PRISMA flowchart.

The PRISMA flow diagram detailing the screening process is presented in Figure 1, and a comprehensive list of excluded studies, along with the reasons for exclusion, is available in Supporting Information S4. Our systematic search identified 4145 records, of which 85 reports on 60 RCTs comprising 22,654 participants met inclusion criteria and were included in the analyses (Figure 1). Of the 60 eligible RCTs, quantitative synthesis was strictly limited to intervention categories with at least three trials (*k* ≥ 3) to ensure statistical stability and interpretability. Consequently, while all 60 trials informed the systematic review, the primary meta‐analyses for self‐harm recurrence at T1, T2, and T3 included 18, 25, and 7 trials, respectively. For suicide death, the number of trials contributing to quantitative synthesis was smaller, with 6 trials at T1, 8 at T2, and 7 at T3 meeting criteria for pooling. This filtering process accounts for the discrepancy between the total number of studies reviewed and those presented in the forest plots. Importantly, risk of bias assessments (RoB 2), certainty of evidence ratings (GRADE), and meta‐regression analyses were conducted independently of this pooling criterion and included all trials that reported the self‐harm outcome. Supporting Information S3 lists all studies included in the systematic review alongside the definitions used for each intervention subgroup.

BIC, defined as an approach that maintains ongoing contact with patients through a combination of in‐person meetings, letters, text messages, telephone calls, or postcards—providing psychoeducation, support, and efforts to reduce isolation and promote alternative coping strategies [16]—was the most frequent intervention, accounting for 41.7% of all interventions. While CBT, case management, and PST interventions were delivered exclusively face‐to‐face (with 8, 4, and 7 in‐person studies, respectively), BIC was notable for its diverse range of delivery formats: in‐person (*n* = 5), remote (*n* = 7), and mixed (*n* = 13). TAU was the most common control condition, utilized in 80% of trials, followed by supportive therapy (4%) (see Supporting Information S3).

Study‐level characteristics are summarized in Supporting Information S5. Most studies were conducted in the UK (23.3%) or the USA (11.7%). The median sample size was 185 (IQR 88–449), and the mean year of study initiation was 2004 (SD 11.2). The mean number of intervention sessions was 7.5 (range 0–58), and the mean follow‐up duration was 15.1 months (SD 12.7). Across studies, the mean participant age was 31.8 years (SD 6.3), the mean proportion of female participants was 66.1% (data missing in 15.0% of studies), and the mean proportion of not married participants was 61.4% (missing in 43.3%). Most studies (65.0%) included participants regardless of suicidal intent, and among those reporting, the mean proportion with a history of repeat self‐harm was 55.9% (missing in 21.7%). For psychiatric diagnoses, the mean study‐level proportion with major depressive disorder was 57.5% (missing in 80.0%), and with borderline personality disorder, 66.9% (missing in 95.0%).

### 4.2 Study Quality and Risk of Bias Assessment

The risk of bias was assessed for 59 of the 60 included trials using the RoB 2 tool. One study was excluded [34] from assessment as it reported only suicide deaths without self‐harm data. Overall, 44.1% of trials were classified as having a low risk of bias, 28.8% as having some concerns, and 25.4% as having a high risk (Supporting Information S6).

Results of publication bias analyses are presented in Supporting Information S7. For new self‐harm at T1, the funnel plot showed good symmetry, and Egger's test did not indicate small‐study effects (*t* = −2.11, df = 16, *p* = 0.0508). For T2, the funnel plot showed mild asymmetry, and Egger's test indicated significant small‐study effects (*t* = −2.62, df = 23, *p* = 0.0152). Accordingly, trim‐and‐fill analysis was performed at T2 and imputed four studies. Publication bias could not be assessed at T3 due to the limited number of studies (*k* = 7).

All subgroup analyses were restricted to comparisons of psychosocial interventions against TAU, since no other intervention type, including pharmacological treatments, had at least three studies reporting the same outcome at a given time point. As a result, forest plots with subgroup analysis were generated only for CBT, BIC, PST, and case management, each compared with TAU.

### 4.3 Psychosocial Interventions Versus Treatment as Usual

**FIGURE 2:**
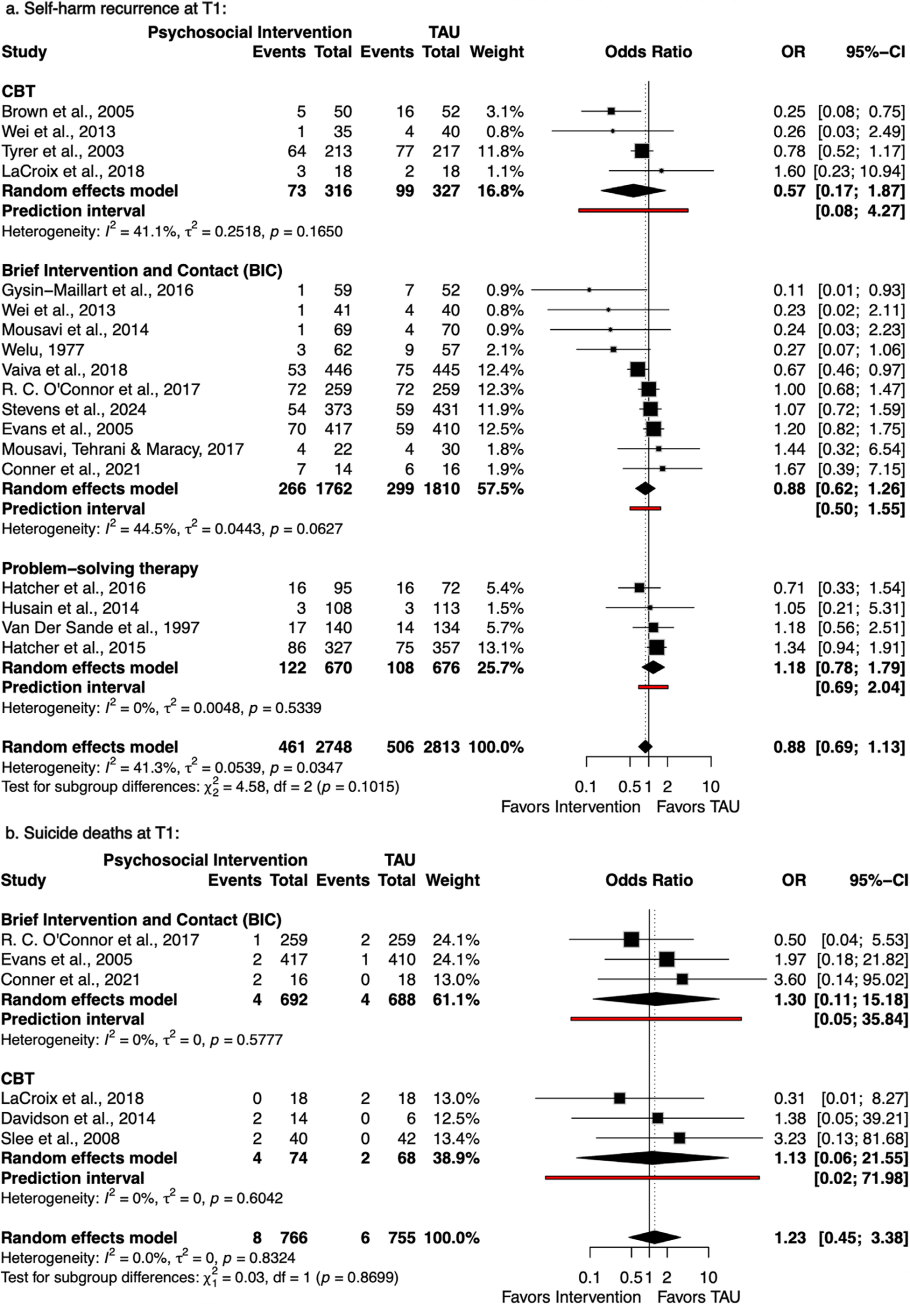
Forest plots of self‐harm recurrence (a) and suicide deaths (b) at T1 (≤ 6 months).

Regarding short‐term follow‐up (T1), 18 [8, 35, 36, 37, 38, 39, 40, 41, 42, 43, 44, 45, 46, 47, 48, 49, 50] and 6 [37, 42, 44, 46, 51, 52] trials reported self‐harm recurrence and suicide deaths, respectively (see Figure 2). For self‐harm, psychosocial interventions showed no statistically significant difference compared to TAU (OR = 0.88; 95% CI: 0.69–1.13; *I* ^2^ = 41.3%). Subgroup analyses by intervention type revealed no significant difference between CBT, BIC, and PST (*p* = 0.10). For suicide deaths, pooled analysis showed no significant impact (OR = 1.23; 95% CI: 0.45–3.38; *I* ^2^ = 0%). Subgroup comparisons between CBT and BIC revealed no differences between BIC and CBT (*p* = 0.87). Prediction intervals of all outcomes and subgroups at T1 included the null.

**FIGURE 3:**
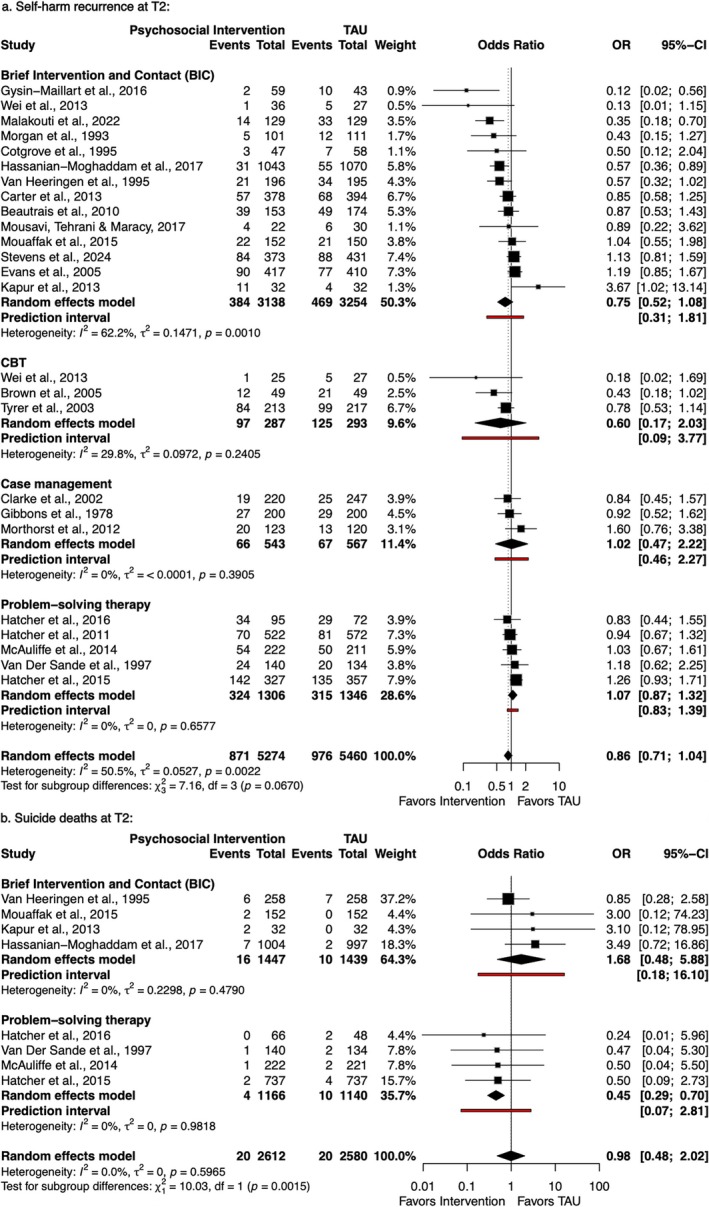
Forest plots of self‐harm recurrence (a) and suicide deaths (b) at T2 (> 6 to 12 months).

Regarding medium‐term follow‐up (T2), 25 [8, 35, 36, 38, 43, 44, 45, 47, 50, 53, 54, 55, 56, 57, 58, 59, 60, 61, 62, 63, 64, 65, 66, 67] and 8 [47, 49, 50, 56, 57, 60, 61, 66] trials reported self‐harm and suicide deaths, respectively (see Figure 3). For self‐harm, psychosocial interventions showed no significant difference versus TAU (OR = 0.86; 95% CI: 0.71–1.04), with significant heterogeneity *I* ^2^ = 50.5% (*p* = 0.0022). Also, subgroup analyses for self‐harm recurrence between BIC, case management, and PST revealed no significant differences (*p* = 0.07). For suicide deaths, pooled estimates showed no significant effect (OR = 0.98; 95% CI: 0.48–2.02; *I* ^2^ = 0%), although with significant subgroup differences between BIC and PST (*p* = 0.0015). PST demonstrated a reduction in suicide deaths when compared to TAU (OR = 0.45; 95% CI: 0.29–0.70; *I* ^2^ = 0%), although the prediction interval was 0.07–2.81. Prediction intervals of other outcomes and subgroups at T2 also included the null.

**FIGURE 4:**
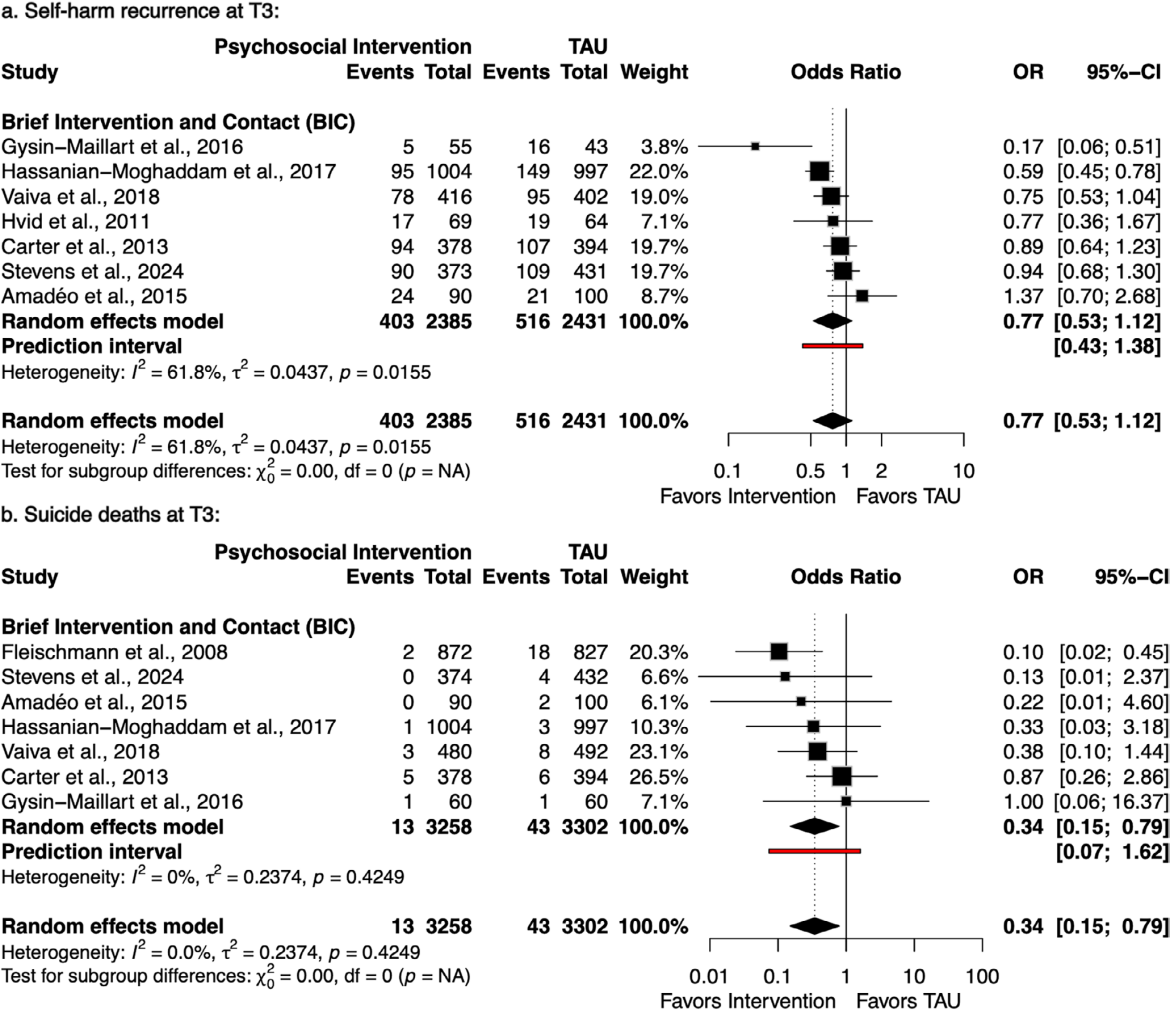
Forest plots of self‐harm recurrence (a) and suicide deaths (b) at T3 (> 12 months).

Regarding long‐term follow‐up (T3), all studies contributing data evaluated the BIC (see Figure 4). For self‐harm, random‐effects analysis of seven trials showed no significant effect (OR = 0.77; 95% CI: 0.53–1.12) [38, 41, 43, 56, 58, 68, 69]. For suicide deaths, seven BIC studies [5, 38, 41, 43, 56, 58, 69] showed significant risk reduction using random‐effect models (OR = 0.34; 95% CI: 0.15–0.79; *I* ^2^ = 0.0%). However, the prediction interval included the null (0.07–1.62).

Sensitivity analyses for suicide deaths that included double‐zero studies (see Supporting Information S8) showed a non‐significant effect at T1. At T2, however, there was a significant reduction in the risk of suicide deaths for PST interventions compared to TAU (OR = 0.53; 95% CI: 0.38–0.75; *I* ^2^ = 0%), although the prediction interval included the null (OR = 0.13–2.12). Notably, at T3, this effect was no longer significant for PST versus TAU (OR = 0.96; 95% CI: 0.83–1.12; *I* ^2^ = 0%). In contrast, significant risk reductions were observed at T3 for BIC (OR = 0.50; 95% CI: 0.35–0.73; *I* ^2^ = 0%), with a prediction interval remaining below the null (0.27–0.94), and for case management interventions (OR = 0.89; 95% CI: 0.83–0.95; *I* ^2^ = 0%) but the prediction interval included the null (0.38–2.08).

### 4.4 Meta‐Regression Analyses

Meta‐regression analyses were performed to examine whether participant, clinical, or intervention characteristics moderated the effectiveness of treatments in reducing self‐harm recurrence (Table 1). No significant moderators were identified at T1 or T3. At T2, selection of participants based on suicidal intent was a significant moderator of intervention effects (OR = 0.57; 95% CI: 0.36–0.89; *p* = 0.015; *R* ^2^ = 37.2%). To further examine this finding, a forest plot stratified by subgroup was created, which confirmed the existence of a statistically significant difference between subgroups (*p* = 0.04), although neither subgroup individually demonstrated a significant effect (see Supporting Information S9). Additionally, mean participant age was also a significant moderator at T2 (OR = 1.05; 95% CI: 1.00–1.09; *p* = 0.03; *R* ^2^ = 82.2%) (see Supporting Information S10 for bubble plots).

**TABLE 1:** Meta‐regression analyses at T1, T2, and T3: Sociodemographic, clinical, and intervention moderators.

Time‐point/variable		*β*	OR	95% CI	SE	*p*	*k*	*I* ^2^	*R* ^2^
T1									
Mean age		0.007	1.007	0.93–1.10	0.043	0.872	14	42.899	0.000
Sex (ref.: female)		0.017	1.018	0.98–1.06	0.019	0.354	17	27.621	14.108
Number of sessions		−0.040	0.961	0.86–1.07	0.057	0.484	14	52.588	0.000
MDD (%)		0.005	1.005	0.93–1.08	0.039	0.904	3	77.291	0.000
Not married (%)		0.010	1.010	0.99–1.03	0.009	0.256	14	0.001	99.990
Repeaters (%)		−0.003	0.997	0.99–1.01	0.004	0.482	14	36.769	0.000
Recruitment beginning (year)		0.013	1.013	0.99–1.04	0.012	0.286	18	40.826	0.000
Suicidal intent description	Regardless of suicidal intent	0.010	1.010	0.80–1.27	0.119	0.934	18	22.289	39.150
	With suicidal intent	−0.330	0.719	0.49–1.07	0.201	0.100	18	22.289	39.150
T2									
Mean age		0.044	1.045	1.00–1.09	0.020	0.030	19	8.263	82.156
Sex (ref.: female)		0.029	1.030	0.99–1.07	0.019	0.112	21	41.170	13.323
Number of sessions		0.035	1.036	0.97–1.11	0.033	0.295	19	49.266	0.000
MDD (%)		−0.009	0.991	0.97–1.01	0.011	0.439	4	19.079	0.000
Not married (%)		0.001	1.001	0.99–1.01	0.006	0.826	15	56.771	0.000
Repeaters (%)		0.002	1.002	0.99–1.01	0.004	0.594	21	49.677	0.000
Recruitment beginning (year)		0.002	1.002	0.99–1.02	0.008	0.793	24	42.979	0.000
Suicidal intent description	Regardless of suicidal intent	−0.077	0.926	0.80–1.07	0.075	0.305	25	30.784	37.218
	With suicidal intent	−0.561	0.571	0.36–0.89	0.229	0.015	25	30.784	37.218
T3									
Mean age		−0.008	0.992	0.91–1.08	0.044	0.862	6	77.973	0.000
Sex (ref.: female)		0.066	1.069	0.99–1.16	0.041	0.109	7	60.824	0.000
Number of sessions		0.022	1.022	0.81–1.29	0.119	0.856	5	81.255	0.000
MDD (%)		Na	Na	Na	Na	Na	Na	Na	Na
Not married (%)		−0.015	0.985	0.95–1.02	0.017	0.381	6	82.387	0.000
Repeaters (%)		−0.021	0.979	0.96–1.00	0.012	0.082	7	55.678	0.000
Recruitment beginning (year)		0.002	1.002	0.95–1.05	0.026	0.949	7	68.926	0.000
Suicidal intent description	Regardless of suicidal intent	−0.155	0.857	0.60–1.21	0.178	0.384	7	65.748	0.000
	With suicidal intent	−0.370	0.691	0.37–1.28	0.313	0.237	7	65.748	0.000

*Note*: Repeaters (%) = percentage of participants with previous self‐harm; reference group for suicidal intent = “regardless of suicidal intent;” MDD (%) = percentage of participants with a diagnosis of Major Depressive Disorder; Not married (%) = percentage of participants who were not married (total minus married); Number of sessions = mean number of sessions delivered per participant during the intervention. β = regression coefficient (log odds ratio); OR = odds ratio; 95% CI = confidence interval; se = standard error; *p* = *p*‐value; *k* = number of studies; *I* ^2^ = heterogeneity; *R* ^2^ = variance explained by moderator(s). “Na” = Not applicable (not enough data to perform regression).

### 4.5 Certainty of Evidence

Supporting Information S11 presents the certainty of evidence for self‐harm recurrence at each time point, limited to comparisons with available forest plots. This was not done for suicide deaths because study‐level RoB2 assessments were not available. CBT was rated very low at T1 and T2, PST was rated low at both T1 and T2, case management was rated low at T2, and BIC was rated low across T1, T2, and T3.

## 5 Discussion

This systematic review and meta‐analysis of 60 RCTs involving 22,654 participants updates the literature by providing critically focused insights into the prevention of repeat self‐harm and suicide following acute presentations. By incorporating eight studies not included in the Cochrane review by Witt et al. [16], including six recently published trials [43, 46, 53, 70, 71, 72], one study reporting longer follow‐up data [73], and the trial by LaCroix et al., which we included despite its exclusion by Witt et al. [16] based on its author correspondence [37], this synthesis expands the available evidence base beyond prior reviews. Importantly, when paired with the strict one‐month inclusion window, these additions allow for a more detailed assessment of intervention effects during the highest risk period for suicide and can inform post‐presentation interventions in clinical services.

PST was associated with lower suicide mortality at medium‐term follow‐up (6–12 months), and BIC was associated with lower suicide mortality at long‐term follow‐up (> 12 months), compared with TAU. However, prediction intervals crossed the null in these analyses, indicating that effects may vary across settings. Across acute‐post presentation trials, effects on repeat self‐harm were consistently small and statistically non‐significant. In addition, RCT‐level patient inclusion based on suicidal intent in the self‐harm act was associated with treatment effectiveness, with larger estimated effects on repeat self‐harm in trials that required suicidal intent at the index episode. Likewise, interventions tended to be more effective in younger samples, with each additional year in mean age associated with about a 4.5% decrease in intervention effectiveness. Notably, these moderators accounted for a substantial share of the variability in treatment effects between studies. All other moderators, including sex, depression, marital status, repeaters, year of recruitment, and number of sessions, showed no significant association with treatment outcomes.

Our long‐term findings are consistent with the review by Riblet et al., which emphasized the effectiveness of BIC in reducing suicide deaths [74]. At that time, this effect was primarily attributed to the WHO‐supported trial by Fleischmann et al. [5]. In contrast, our review incorporates more recent trials that tested variations of BIC across diverse populations, including those conducted in Australia and France [41, 43]. The sustained effectiveness of BIC in reducing suicide mortality over long‐term follow‐up suggests that timely therapeutic contact may be a key determinant of outcomes in the aftermath of self‐harm. This effect likely reflects the distinctive vulnerability and clinical relevance of the immediate post‐self‐harm period. BIC's effectiveness likely stems from its alignment with these psychological realities: it requires minimal cognitive demands, maintains a non‐judgmental human connection during peak vulnerability, and directly addresses the thwarted belongingness and perceived burdensomeness central to Joiner's interpersonal theory of suicide [38]. Importantly, while the pooled effect for BIC was statistically significant, the prediction interval included the null value, suggesting that the effect may not be consistent across all settings or future studies. This highlights the need for cautious interpretation and for further research to establish the reliability and generalizability of BIC's impact on suicide death prevention. Additionally, methodological differences between studies evaluating BIC and those testing more structured interventions could contribute to observed differences in efficacy. The absence of direct comparative trials between BIC and structured psychotherapies further limits our ability to determine whether any differential effectiveness reflects true intervention superiority or simply methodological variation. Accordingly, future research should prioritize head‐to‐head comparisons between brief contact interventions and structured psychotherapies to clarify their relative effectiveness, ensure the robustness of these findings, and guide optimal intervention sequencing in the post‐self‐harm period.

In this meta‐analysis, PST was associated with a 55% reduction in suicide deaths at medium‐term follow‐up (6–12 months) compared to TAU, suggesting potential for suicide prevention following acute presentations. This aligns with longstanding evidence that suicidal individuals frequently present with impaired problem‐solving, reduced cognitive flexibility, and limited capacity to generate alternative solutions [75, 76]. However, despite the pooled data suggesting a beneficial effect of PST, findings from individual trials were mixed and often non‐significant. The culturally adapted *Te Ira Tangata* trial for Māori populations showed a significant reduction in self‐harm re‐presentations at 3 months (10.4% vs. 18%, *p* = 0.04), but not at 12 months [47]. Similarly, the ACCESS trial, which offered PST alongside brief contact postcards, failed to show significant differences in repetition rates, partly due to limited engagement—only half of the participants received PST [50]. McAuliffe et al. evaluated group‐based PST and found no superiority over usual care in reducing repeated self‐harm at 12 months [66]. Lastly, van der Sande et al. compared intensive psychosocial care incorporating PST to routine care and found no differences in suicide reattempt rates (17% vs. 15%) [77]. Although individual trial results were limited, PST appears to hold promise for suicide death prevention when tailored to the appropriate clinical and cultural context. Future research should clarify which elements, such as specific psychotherapeutic components, cultural adaptation, early engagement, or delivery setting, are most critical to enhancing its effectiveness.

Our meta‐analysis found no significant effect of CBT on repeat suicide attempts at either short‐term (T1) or medium‐term (T2) follow‐up. This is in contrast to earlier high‐impact trials such as Brown et al., where 10 sessions of cognitive therapy reduced suicide reattempts by 50% over 18 months compared to enhanced usual care [8]. However, other included trials showed more variable results. Tyrer et al., in the POPMACT study, found no significant reduction in repeat self‐harm with manual‐assisted CBT compared to TAU (39% vs. 46%), despite cost‐effectiveness advantages [36]. In the Wei et al. trial in China, CBT did not significantly reduce reattempt rates, partly due to poor treatment uptake, as only 6.1% of participants assigned to CBT actually received therapy [35]. Trials with longer follow‐up periods (T2) also reported inconsistent findings. Donaldson et al. observed reductions in suicidal ideation in adolescents at 3 and 6 months, but no differences between CBT and a control supportive therapy group [78]. Sinyor et al. showed that brief CBT was associated with significantly fewer weeks of self‐harm (11%) compared to control supportive psychotherapy (30%), although sample size and retention were limited [7]. Additionally, Lin et al. found that brief CBT plus case management approximately halved the odds of suicide attempts at 6 months, but this effect was not maintained at 12 months [79]. These findings suggest that CBT may offer some short‐term benefit under specific conditions, but limited treatment exposure and variable comparison conditions (e.g., supportive therapy, case management) likely attenuated the pooled effect in our analysis.

The identification of suicidal intent as an effect modifier highlights critical methodological and conceptual challenges in self‐harm research. While self‐harm with and without suicidal intent may reflect partially distinct underlying mechanisms, these behaviors are frequently indistinguishable at the point of acute clinical presentation, particularly in emergency settings where intent is often ambivalent, fluctuating, or retrospectively reconstructed [80, 81]. This clinical reality supports the rationale for a unified clinical framework. At the same time, our findings indicate that suicidal intent retains prognostic and therapeutic relevance. Interventions were approximately 43% more effective in reducing repeat self‐harm in studies that restricted inclusion to individuals with explicit suicidal intent, as demonstrated by meta‐regression analyses. The field's tendency to combine populations with potentially distinct underlying mechanisms—such as those who self‐harm with clear suicidal intent versus those engaging in non‐suicidal self‐injury—may therefore contribute to the modest effects observed in RCTs of psychosocial interventions for repeat self‐harm [13]. Although our subgroup analyses found larger effect sizes in studies recruiting participants with suicidal intent compared to those with mixed or non‐suicidal intent populations, the confidence intervals overlapped and did not reach statistical significance. This likely reflects limited statistical power within subgroups. However, this significant association suggests a genuine difference in treatment responsiveness patterns that may be masked when populations are aggregated. Consistent with these findings, emerging neurocognitive evidence indicates that suicidal and non‐suicidal self‐harm may represent distinct phenotypes with different underlying neural mechanisms, cognitive patterns, and treatment needs [82, 83]. Nonetheless, several clinical factors limit a definitive conclusion. First, the assessment of suicidal intent remains methodologically challenging, as intent exists on a continuum rather than as a binary construct. Individuals frequently experience ambivalent feelings toward death during self‐harm episodes, oscillating between a full desire for death and seeking psychological pain relief, and there is ongoing debate about whether these represent two distinct phenotypes [84, 85, 86, 87]. Second, intent can fluctuate dynamically and may be influenced by retrospective reporting biases or assessment biases, particularly in emergency settings [80, 88].

Furthermore, our findings regarding mean age suggest that psychosocial interventions may be less effective for older adults, highlighting the need for age‐sensitive approaches and potentially distinct intervention strategies across the lifespan. Prior evidence indicates that older adults are less likely to receive comprehensive suicide risk screening and management interventions, such as safety planning and lethal means counseling, which may contribute to poorer outcomes in this group [89]. Moreover, older adults bear the highest mortality rates due to suicide, and the need for targeted, high‐quality research in late‐life populations has already been emphasized in the literature [90, 91]. Our findings reinforce the importance of this research gap and indicate that the need for tailored interventions is continuous across the lifespan. This suggests that intervention strategies for self‐harm prevention should be adapted according to the age profile of the population, ensuring that the specific needs of different age groups are adequately addressed.

These findings have important implications for clinical practice guidelines. While recommendations from NICE emphasize structured psychotherapies such as CBT and DBT [17], our results suggest that during the acute post‐presentation window, brief and scalable interventions, particularly BIC and PST, may offer greater clinical utility for immediate suicide risk reduction. Notably, when applying our temporal inclusion criterion (enrollment ≤ 1 month following self‐harm), DBT was represented by too few trials to permit quantitative pooling at any follow‐up interval. Although landmark trials such as those by Mehlum et al. and McCauley et al. have yielded promising results, and high‐certainty reviews have established DBT as a first‐line intervention for adolescents and individuals with borderline personality disorder [9, 17, 92, 93], these trials typically enrolled participants well beyond the acute post‐presentation period. This represents a critical evidence gap: DBT remains untested mainly in acute clinical settings where intervention must be initiated shortly after a self‐harm episode. Future trials should evaluate DBT effectiveness with enrollment restricted to the immediate post‐presentation window and with outcomes assessed at short‐, mid‐, and long‐term intervals.

Additionally, pharmacological evidence for the hours and days following a self‐harm presentation remains scant and methodologically fragile. In the Cochrane synthesis of seven trials conducted during crisis periods, only one small study of a depot antipsychotic suggested reduced repeat self‐harm, but its limited sample (*N* = 30) and high overall risk of bias preclude firm conclusions [10]. Conventional antidepressants lack dedicated research in suicidal crisis settings and are further limited by depression trials that routinely exclude high‐risk patients, leaving a substantial evidence void for emergency applications [94, 95]. Glutamatergic agents such as ketamine and intranasal esketamine appear to rapidly suppress suicidal ideation within 4–24 h in a handful of placebo‐controlled trials, yet these studies are typically underpowered for behavioral endpoints, employ heterogeneous dosing schedules, and provide limited follow‐up [11, 96, 97]. Lithium, which remains possibly the most consistently supported drug for long‐term suicide prevention in mood disorders, again demonstrated only non‐significant trends in the latest 15‐trial meta‐analysis of acute and maintenance settings [98]. In aggregate, the existing data do not sufficiently support any standalone pharmacological recommendation for the immediate post‐attempt period.

Beyond efficacy, psychosocial and pharmacological interventions present fundamentally different implementation challenges in acute care settings. Brief psychosocial strategies, such as BIC, are low‐cost, require minimal training, and can be initiated immediately following emergency presentation, whereas intensive psychotherapies like CBT and DBT demand specialized training, sustained therapeutic contact, and adequate workforce capacity, which may limit rapid deployment. Pharmacological interventions face distinct barriers: most antidepressants have a delayed onset of action, limiting their utility during the immediate high‐risk window; rapid‐acting agents such as ketamine require specialized settings and monitoring; and medication adherence post‐discharge is often poor. These realities underscore that intervention selection in acute care must balance efficacy, feasibility, safety, and local healthcare capacity.

The temporal dynamics of intervention effects offer mechanistic insights. The emergence of significant effects only at medium‐ and long‐term follow‐up suggests that brief contacts may initiate cascading protective processes rather than providing immediate symptom relief. Notably, at short‐term follow‐up (≤ 6 months), point estimates for several psychosocial interventions trended toward increased risk. Although confidence intervals consistently crossed the null—precluding conclusions of iatrogenic harm—this pattern highlights the absence of immediate protective benefit. These findings carry two clinical implications: first, they reinforce the need for heightened vigilance during the early post‐discharge period; second, they suggest that mechanisms underlying interventions such as PST and BIC may require time to consolidate before translating into measurable reductions in behavioral risk. This pattern aligns with recent work on crisis response planning, an intervention that helps suicidal individuals recognize early indicators of an emerging crisis and personalized strategies they can use to self‐regulate, obtain support from social connections, and enhance self‐efficacy for crisis management [99, 100]. In two previous RCTs of crisis response planning, suicidal patients who received the intervention showed significantly faster reductions in suicidal ideation within 1 month, and significant reductions in suicide attempts emerged after 6 months [100, 101]. The delayed effect may also reflect the episodic nature of suicidal crises; brief contacts provide a safety net that becomes most relevant when individuals face subsequent stressors months after the index episode. Yet another possibility relates to suicidal ideation's continuous nature relative to suicidal behavior's discrete nature. Because it is easier statistically to assess change in continuous constructs, identifying treatment effects on suicide attempt risk in the short term can be difficult. This understanding challenges trial designs that focus on short‐term outcomes, suggesting that the field may have underestimated the effectiveness of interventions by prioritizing immediate effects over sustained ones.

Several limitations warrant consideration. First, despite identifying 60 trials, many intervention subgroups contained fewer than three studies, precluding forest plots and limiting certain subgroup analyses. Second, deviations from our PROSPERO protocol occurred: intervention categorization was modified during data extraction to enhance clinical interpretability, and secondary outcomes (suicidal ideation, depressive symptoms) were deferred to a separate report, with suicide death prioritized given its clinical relevance. Third, even at the longest follow‐up, pooled analyses included only 56 suicide deaths (43 TAU vs. 13 intervention), highlighting the persistent challenge of detecting differences in this rare outcome; findings for PST and BIC should therefore be interpreted cautiously. Fourth, methodological constraints include potential bias from self‐reported outcomes, limited generalizability due to our one‐month enrollment window, incomplete reporting of key moderators (substance use, treatment adherence), and high dropout rates that may have undermined interventions requiring sustained engagement. Moreover, these methodological differences are likely to interact with participants' baseline risk profiles. Trials employing broad self‐harm definitions may capture heterogeneous samples spanning non‐suicidal self‐injury to high‐lethality suicide attempts, each associated with distinct recurrence trajectories and treatment responsiveness. Similarly, variation in follow‐up periods may differentially affect outcome detection depending on baseline severity, with high‐risk individuals more likely to experience early recurrence. This interaction between design‐level and participant‐level heterogeneity may compound the observed variability in pooled estimates and partly account for the limited ability to detect intervention effects on repeat self‐harm. Fifth, study‐level subgroup analyses are observational and potentially confounded by systematic differences between trial populations. Finally, the evidence base is geographically skewed toward high‐income settings (UK, US) with limited representation from low‐ and middle‐income countries, and overrepresentation of female participants may limit generalizability to males, who exhibit distinct risk profiles.

Our findings suggest that brief, scalable post‐presentation strategies—particularly BIC and PST—may have clinical value, including potential associations with lower suicide mortality at medium‐ and long‐term follow‐up. At the same time, effects on repeat self‐harm were small and not statistically significant, highlighting the limitations of this outcome in heterogeneous acute post‐presentation samples. These results support the pragmatic use of early low‐burden interventions as complements to longer‐term treatments when indicated and emphasize priorities for future adequately powered trials designed for the immediate post‐presentation window, including careful attention to rare outcomes and implementation context.

## Author Contributions

R.F.D., C.J.B., and E.C.M. contributed to the conceptualization and methodology of this review. G.P.R. and R.F.D. designed the study and drafted the initial manuscript. G.P.R. and L.B. conducted the screening and eligibility process, with R.F.D. providing guidance on disagreements. G.P.R., K.L.R., and B.B.F.D. performed data extraction, and G.P.R. and L.C.F. carried out the analyses. C.J.B. and E.C.M. provided critical input for the interpretation of results and revisions of the manuscript. R.F.D. supervised the project. All authors reviewed and approved the final version of the manuscript.

## Funding

G.P.R. received a scholarship from São Paulo Research Foundation (FAPESP) (process number 2023/14905‐7); R.F.D. received a scholarship from FAPESP (process number 2024/10058‐0). The Article Processing Charge for the publication of this research was funded by the Coordenação de Aperfeiçoamento de Pessoal de Nível Superior—Brasil (CAPES) (ROR identifier: 00x0ma614).

## Ethics Statement

The authors have nothing to report.

## Consent

The authors have nothing to report.

## Conflicts of Interest

The authors declare no conflicts of interest.

## Supporting information


**Supporting Information: S1.** PRISMA 2020 Checklist.
**Supporting Information: S2**. Boolean search strategy.
**Supporting Information: S3** Studies included in the systematic review.
**Supporting Information: S4** List of excluded studies.
**Supporting Information: S5** Characteristics of randomized controlled trials included in our review.
**Supporting Information: S6** RoB 2 Summary by Study and Domain.
**Supporting Information: S7** Publication bias: funnel plots with trim‐and‐fill.
**Supporting Information: S8** New Suicide Deaths at each time‐point, including double‐zero studies.
**Supporting Information: S9** Psychosocial Interventions vs. TAU: New Self‐Harm at T2 by Suicidal Intent (Regardless vs. With suicidal intent).
**Supporting Information: S10** Bubble plots for suicidal intent and mean age at T2.
**Supporting Information: S11** GRADE evidence tables.

## Data Availability

All data used in this systematic review and meta‐analysis are derived from published studies and publicly available trial registries. Extracted data supporting the findings of this study are available from the corresponding author upon reasonable request.
