# Supplementary material for: Acute‐Phase Interventions After Self‐Harm for Preventing Suicide and Recurrence: A Systematic Review and Meta‐Analysis
Source: Acta Psychiatr Scand. 2026 Mar 9;153(6):610–26. doi: 10.1111/acps.70085 (PMC13124320; doi:10.1111/acps.70085)
Supplement: Supplementary file 1 — Supporting Information: S1. PRISMA 2020 Checklist. Supporting Information: S2. Boolean search strategy. Supporting Information: S3 Studies included in the systematic review. Supporting Information: S4 List of excluded studies. Supporting Information: S5 Characteristics of randomized controlled trials included in our review. Supporting Information: S6 RoB 2 Summary by Study and Domain. Supporting Information: S7 Publication bias: funnel plots with trim‐and‐fill. Supporting Information: S8 New Suicide Deaths at each time‐point, including double‐zero studies. Supporting Information: S9 Psychosocial Interventions vs. TAU: New Self‐Harm at T2 by Suicidal Intent (Regardless vs. With suicidal intent). Supporting Information: S10 Bubble plots for suicidal intent and mean age at T2. Supporting Information: S11 GRADE evidence tables. [file ACPS-153-610-s001.docx]

**Supplementary Material 1.** PRISMA 2020 Checklist

| **Section and Topic** | **Item #** | **Checklist item** | **Location where item is reported** |
| --- | --- | --- | --- |
| **TITLE** | | |  |
| Title | 1 | Identify the report as a systematic review. | p. 1 |
| **ABSTRACT** | | |  |
| Abstract | 2 | See the PRISMA 2020 for Abstracts checklist. | p. 3-4 |
| **INTRODUCTION** | | |  |
| Rationale | 3 | Describe the rationale for the review in the context of existing knowledge. | p. 5-7 |
| Objectives | 4 | Provide an explicit statement of the objective(s) or question(s) the review addresses. | p. 7 |
| **METHODS** | | |  |
| Eligibility criteria | 5 | Specify the inclusion and exclusion criteria for the review and how studies were grouped for the syntheses. | p. 8-9/11-12 |
| Information sources | 6 | Specify all databases, registers, websites, organisations, reference lists and other sources searched or consulted to identify studies. Specify the date when each source was last searched or consulted. | p. 8 |
| Search strategy | 7 | Present the full search strategies for all databases, registers and websites, including any filters and limits used. | p. 8 (SM1) |
| Selection process | 8 | Specify the methods used to decide whether a study met the inclusion criteria of the review, including how many reviewers screened each record and each report retrieved, whether they worked independently, and if applicable, details of automation tools used in the process. | p. 8-9 |
| Data collection process | 9 | Specify the methods used to collect data from reports, including how many reviewers collected data from each report, whether they worked independently, any processes for obtaining or confirming data from study investigators, and if applicable, details of automation tools used in the process. | p. 9 |
| Data items | 10a | List and define all outcomes for which data were sought. Specify whether all results that were compatible with each outcome domain in each study were sought (e.g. for all measures, time points, analyses), and if not, the methods used to decide which results to collect. | p. 9-10 |
|  | 10b | List and define all other variables for which data were sought (e.g. participant and intervention characteristics, funding sources). Describe any assumptions made about any missing or unclear information. | p. 9 |
| Study risk of bias assessment | 11 | Specify the methods used to assess risk of bias in the included studies, including details of the tool(s) used, how many reviewers assessed each study and whether they worked independently, and if applicable, details of automation tools used in the process. | p. 10-11 |
| Effect measures | 12 | Specify for each outcome the effect measure(s) (e.g. risk ratio, mean difference) used in the synthesis or presentation of results. | p. 11-12 |
| Synthesis methods | 13a | Describe the processes used to decide which studies were eligible for each synthesis (e.g. tabulating the study intervention characteristics and comparing against the planned groups for each synthesis (item #5)). | p. 11-12 |
|  | 13b | Describe any methods required to prepare the data for presentation or synthesis, such as handling of missing summary statistics, or data conversions. | p. 11-12 |
|  | 13c | Describe any methods used to tabulate or visually display results of individual studies and syntheses. | p. 11-12 |
|  | 13d | Describe any methods used to synthesize results and provide a rationale for the choice(s). If meta-analysis was performed, describe the model(s), method(s) to identify the presence and extent of statistical heterogeneity, and software package(s) used. | p. 11-12 |
|  | 13e | Describe any methods used to explore possible causes of heterogeneity among study results (e.g. subgroup analysis, meta-regression). | p. 11-12 |
|  | 13f | Describe any sensitivity analyses conducted to assess robustness of the synthesized results. | p. 11-12 |
| Reporting bias assessment | 14 | Describe any methods used to assess risk of bias due to missing results in a synthesis (arising from reporting biases). | p. 11 |
| Certainty assessment | 15 | Describe any methods used to assess certainty (or confidence) in the body of evidence for an outcome. | p. 13 |
| **RESULTS** | | |  |
| Study selection | 16a | Describe the results of the search and selection process, from the number of records identified in the search to the number of studies included in the review, ideally using a flow diagram. | p. 13 |
|  | 16b | Cite studies that might appear to meet the inclusion criteria, but which were excluded, and explain why they were excluded. | p. 13 (SM3) |
| Study characteristics | 17 | Cite each included study and present its characteristics. | p. 13 (SM2) |
| Risk of bias in studies | 18 | Present assessments of risk of bias for each included study. | p. 14 (SM5) |
| Results of individual studies | 19 | For all outcomes, present, for each study: (a) summary statistics for each group (where appropriate) and (b) an effect estimate and its precision (e.g. confidence/credible interval), ideally using structured tables or plots. | p. 15-16 |
| Results of syntheses | 20a | For each synthesis, briefly summarise the characteristics and risk of bias among contributing studies. | p. 14 |
|  | 20b | Present results of all statistical syntheses conducted. If meta-analysis was done, present for each the summary estimate and its precision (e.g. confidence/credible interval) and measures of statistical heterogeneity. If comparing groups, describe the direction of the effect. | p. 15 |
|  | 20c | Present results of all investigations of possible causes of heterogeneity among study results. | p. 15-16 |
|  | 20d | Present results of all sensitivity analyses conducted to assess the robustness of the synthesized results. | p. 16 |
| Reporting biases | 21 | Present assessments of risk of bias due to missing results (arising from reporting biases) for each synthesis assessed. | p. 14 (SM6) |
| Certainty of evidence | 22 | Present assessments of certainty (or confidence) in the body of evidence for each outcome assessed. | p. 17 (SM11) |
| **DISCUSSION** | | |  |
| Discussion | 23a | Provide a general interpretation of the results in the context of other evidence. | p. 17 |
|  | 23b | Discuss any limitations of the evidence included in the review. | p. 22-23 |
|  | 23c | Discuss any limitations of the review processes used. | p. 22-23 |
|  | 23d | Discuss implications of the results for practice, policy, and future research. | p. 23-24 |
| **OTHER INFORMATION** | | |  |
| Registration and protocol | 24a | Provide registration information for the review, including register name and registration number, or state that the review was not registered. | p. 1 |
|  | 24b | Indicate where the review protocol can be accessed, or state that a protocol was not prepared. | p. 7 |
|  | 24c | Describe and explain any amendments to information provided at registration or in the protocol. | p. 23 |
| Support | 25 | Describe sources of financial or non-financial support for the review, and the role of the funders or sponsors in the review. | p. 1 |
| Competing interests | 26 | Declare any competing interests of review authors. | p. 1 |
| Availability of data, code and other materials | 27 | Report which of the following are publicly available and where they can be found: template data collection forms; data extracted from included studies; data used for all analyses; analytic code; any other materials used in the review. | p. 2 |

**Supplementary Material 2**. Boolean search strategy

| **Database** | **Advanced search** |
| --- | --- |
| Pubmed | (suic*[Title/Abstract] OR parasuicide[Title/Abstract] OR self-harm*[Title/Abstract] OR self-injur*[Title/Abstract] OR self-viol*[Title/Abstract] OR self-pois*[Title/Abstract]) AND (prevent*[Title/Abstract] OR “reduce”[Title/Abstract] OR treat*[Title/Abstract] OR therapy*[Title/Abstract] OR intervent*[Title/Abstract] OR acute*[Title/Abstract] OR repeat*[Title/Abstract] OR recurr*[Title/Abstract] OR repet*[Title/Abstract] OR manage*[Title/Abstract] OR “care”[Title/Abstract]) AND (“emergency”[Title/Abstract] OR “urgency”[Title/Abstract] OR hospital*[Title/Abstract] OR “medical centers”[Title/Abstract] OR “medical center”[Title/Abstract] OR “infirmary”[Title/Abstract] OR “discharge”[Title/Abstract] OR department*[Title/Abstract]) AND (randomized controlled trial[pt] OR controlled clinical trial[pt] OR randomized[title/abstract] OR placebo[title/abstract] OR clinical trials as topic[mesh:noexp] OR randomly[title/abstract] OR trial[title]  NOT (animals[mh] NOT humans [mh])) |
| Embase | (suic*:ab,ti OR parasuicide:ab,ti OR 'self-harm*':ab,ti OR 'self-injur*':ab,ti OR 'self-viol*':ab,ti OR 'self-pois*':ab,ti) AND (prevent*:ab,ti OR 'reduce':ab,ti OR treat*:ab,ti OR therapy*:ab,ti OR intervent*:ab,ti OR acute*:ab,ti OR repeat*:ab,ti OR recurr*:ab,ti OR repet*:ab,ti OR manage*:ab,ti OR 'care':ab,ti) AND ('emergency':ab,ti OR 'urgency':ab,ti OR hospital*:ab,ti OR 'medical center':ab,ti OR 'medical centers':ab,ti OR 'infirmary':ab,ti OR 'discharge':ab,ti OR department*:ab,ti) AND (randomized AND controlled AND trial OR (controlled AND clinical AND trial) OR randomized OR placebo OR randomly OR trial) |
| PsycINFO | (title: randomized controlled trial OR title: controlled clinical trial OR title: randomized OR title: placebo OR title: clinical trials as topic OR title: randomly OR title: trial) NOT (title: animals NOT title: humans) AND ((title: suic* OR title: parasuicide OR title: self-harm* OR title: self-viol* OR title: self-pois* OR (abstract: suic* OR abstract: parasuicide OR abstract: self-harm* OR  abstract: self-viol* OR abstract: self-pois*)) AND ((title: prevent* OR title: reduce OR title: treat* OR title: therapy OR title: intervent* OR title: acute OR title: repeat* OR title: recurr* OR title: repet* OR title: manage* OR title: care) OR (abstract: prevent* OR abstract: reduce OR abstract: treat* OR abstract: therapy OR abstract: intervent* OR abstract: acute OR abstract: repeat* OR abstract: recurr* OR abstract: repet* OR abstract: manage* OR abstract: care)) AND ((title: emergency OR title: urgency OR title: hospital* OR title: discharge OR title: ‘medical center’ OR title: ‘medical centers’ OR title: infirmary OR title: department*) OR  (abstract: emergency OR abstract: urgency OR abstract: hospital* OR abstract: discharge OR abstract: ‘medical center’ OR abstract: ‘medical centers’ OR abstract: infirmary OR abstract: department*)) |
| WHO ICTRP | (suic* OR parasuicide OR 'self-harm*' OR 'self-injur*' OR 'self- viol*' OR 'self-pois*') AND (prevent* OR 'reduce' OR treat* OR therapy* OR intervent* OR acute* OR repeat* OR recurr* OR repet* OR manage* OR 'care') AND ('emergency' OR 'urgency' OR hospital* OR 'medical center' OR 'medical centers' OR 'infirmary' OR 'discharge' OR department*) |
| ClinicalTrials.gov | (suic* OR parasuicide OR 'self-harm*' OR 'self-injur*' OR 'self-viol*' OR 'self-pois*') AND (prevent* OR 'reduce' OR treat* OR therapy* OR intervent* OR acute* OR repeat* OR recurr* OR repet* OR manage* OR 'care') AND ('emergency' OR 'urgency' OR hospital* OR 'medical center' OR 'medical centers' OR 'infirmary' OR 'discharge' OR department*) |

**Supplementary Material 3.** Studies included in the systematic review.

| Study | Control | Intervention(s) | Delivery | Age, y | | Repeaters (%) | MDD (%) | Female (%) | Suicidal intent |
| --- | --- | --- | --- | --- | --- | --- | --- | --- | --- |
|  |  |  |  | Mean | SD |  |  |  |  |
| Stevens et al., 2024 | TAU | BIC | Remote (online/SMS) | na | na | 31.1 | na | 64.5 | Regardless |
| Law et al., 2023 | TAU | BIC | Remote (online) / Mixed | 24.9 | 9.0 | na | na | na | Regardless |
| Malakouti et al., 2022 | TAU | BIC | Mixed | na | na | 43.4 | na | 65.1 | With |
| R. C. O'Connor et al., 2022 | TAU | Safety planning | Mixed | 37.0 | 14.8 | 95.0 | na | 62.5 | With |
| Arvilommi et al., 2022 | Supportive therapy | BIC | Mixed | 32.0 | 12.4 | 60.6 | 69.4 | 72.2 | With |
| Conner et al., 2021 | TAU | BIC | Mixed | 38.4 | 17.8 | 50.0 | 83.0 | 63.0 | With |
| Lin et al., 2020 | Case management | CBT | In person | na | na | 69.0 | 37.3 | 74.7 | With |
| Cottrell et al., 2020 | TAU | Family intervention | In person | na | na | 100.0 | na | 88.0 | Regardless |
| Sinyor et al., 2020 | Supportive therapy | CBT | In person | 18.0 | 3.2 | na | 91.7 | 58.0 | Regardless |
| Vaiva et al., 2018 | TAU | BIC | In person | 38.1 | 13.1 | 45.1 | 42.3 | 63.6 | With |
| S. S. O'Connor et al., 2020 | TAU | Psychodynamic | In person | 42.0 | 2.7 | na | na | 48.0 | With |
| LaCroix et al., 2018 | TAU | CBT | In person | 33.0 | 10.8 | na | na | 27.8 | With |
| Hassanian-Moghaddam et al., 2017 | TAU | BIC | Remote (postcards) | 24.0 | 8.1 | 33.0 | na | 66.0 | Regardless |
| R. C. O'Connor et al., 2017 | TAU | BIC | In person | 36.1 | 12.8 | 100.0 | na | 63.0 | With |
| Mousavi, Tehrani & Maracy, 2017 | TAU | BIC | Mixed | na | na | 100.0 | na | 73.3 | With |
| Gysin-Maillart et al., 2016 | TAU | BIC | Mixed | 39.2 | 14.6 | 57.0 | na | 50.0 | With |
| Hatcher et al., 2016 | TAU | PST | In person | 33.2 | 11.4 | 55.6 | na | 59.7 | Regardless |
| Mousavi et al., 2016 | BIC | BIC | Remote (phone call) | 27.1 | 7.8 | 100.0 | na | 93.0 | With |
| Andreoli et al., 2016 | TAU | Psychodynamic | In person | 32.7 | 10.9 | 70.0 | 86.7 | 86.7 | Regardless |
| Armitage et al., 2016* | TAU | Supportive Therapy and Help Sheet | In person | 28.3 | 11.4 | na | na | 71.0 | Regardless |
| Hvid et al., 2011 | TAU | BIC | Mixed | 37.4 | 16.4 | 39.0 | na | 72.0 | With |
| Grimholt et al., 2015 | TAU | Supportive therapy | In person | 40.0 | na | 48.1 | na | 72.4 | Regardless |
| Hatcher et al., 2015 | TAU | PST | Mixed | 36.2 | 14.2 | 54.3 | na | 70.6 | Regardless |
| Amadéo et al., 2015 | TAU | BIC | Mixed | 31.5 | na | 37.0 | na | 64.0 | Regardless |
| Mouaffak et al., 2015 | TAU | BIC | Remote (letters) | 38.6 | 13.3 | 47.9 | 27.1 | 73.5 | With |
| Husain et al., 2014 | TAU | PST | In person | 23.1 | 5.3 | 3.5 | na | 67.3 | Regardless |
| Kawanishi et al., 2014 | TAU | Case management | In person | 41.7 | 15.2 | 48.2 | na | 55.0 | Regardless |
| Davidson et al., 2014 | TAU | CBT | In person | na | na | na | na | na | Regardless |
| McAuliffe et al., 2014 | TAU | PST | In person | 33.6 | 12.2 | 63.0 | na | 65.0 | Regardless |
| Mousavi et al., 2014 | TAU | BIC | Remote (phonecall) | na | na | 100.0 | na | 54.3 | Regardless |
| Kapur et al., 2013 | TAU | BIC | Mixed | na | na | 53.0 | na | na | Regardless |
| Wei et al., 2013** | TAU | BIC and CBT | Remote (telephone) / In person | 32.1 | 13.9 | na | na | 75.3 | Regardless |
| Carter et al., 2013 | TAU | BIC | Mixed | 35.5 | 14.6 | 17.0 | na | 74.0 | Regardless |
| Morthorst et al., 2012 | TAU | Case management | In person | 30.5 | 12.1 | 53.0 | na | 73.0 | Regardless |
| Ougrin et al., 2011 | Supportive therapy | Therapeutic Assessment | Delivery | 15.5 | 1.2 | 54.0 | na | 80.0 | Regardless |
| Hatcher et al., 2011 | TAU | PST |  | 34.2 | 13.2 | 43.0 | na | 69.0 | Regardless |
| Beautrais et al., 2010 | TAU | BIC | Remote (online/SMS) | 33.9 | na | 19.4 | na | 62.3 | Regardless |
| Fleischmann et al., 2008 | TAU | BIC | Remote (online) / Mixed | na | na | 20.1 | na | 56.5 | Regardless |
| Slee et al., 2008 | TAU | CBT | Mixed | 25.4 | 6.5 | na | na | 91.0 | Regardless |
| Hallahan et al., 2007 | Placebo | Omega 3 | Mixed | 30.7 | na | 100.0 | na | 63.0 | Regardless |
| Donaldson et al., 2005 | Supportive therapy | CBT | Mixed | na | na | 44.0 | 31.0 | na | With |
| Evans et al., 2005 | TAU | BIC | Mixed | 33.8 | 13.1 | 49.3 | na | 63.0 | Regardless |
| Brown et al., 2005 | TAU | CBT | In person | 34.9 | 10.5 | 71.7 | 75.0 | 61.7 | With |
| Tyrer et al., 2003 | TAU | CBT | In person | 32.0 | na | 100.0 | na | 68.0 | Regardless |
| Bennewith et al., 2002 | TAU | Guideline for GP | In person | 32.8 | 13.0 | 11.4 | na | 57.3 | Regardless |
| Clarke et al., 2002 | TAU | Case management | In person | 32.0 | na | 49.0 | 59.0 | 55.1 | Regardless |
| Guthrie et al., 2001 | TAU | Psychodynamic | In person | 31.1 | na | 27.7 | na | 54.1 | Regardless |
| Battaglia et al., 1999 | Typical antipsychotic | Typical antipsychotic | In person | 31.2 | 8.2 | 100.0 | na | 44.4 | With |
| Harrington et al., 1998 | TAU | Family intervention | Remote (postcards) | 14.6 | 1.1 | na | 69.0 | 90.0 | Regardless |
| Verkes et al., 1998 | Placebo | SSRI | In person | 37.1 | 13.0 | 100.0 | na | 56.0 | With |
| Van Der Sande et al., 1997 | TAU | PST | Mixed | 36.8 | 14.6 | 43.8 | na | 65.7 | Regardless |
| Cotgrove et al., 1995 | TAU | BIC | Mixed | na | na | na | na | na | Regardless |
| Van Heeringen et al., 1995 | TAU | BIC | In person | 33.8 | na | 32.6 | na | 52.3 | Regardless |
| McLeavey et al., 1994 | PST | PST | Remote (phone call) | 25.3 | 8.1 | 25.0 | na | 70.0 | Regardless |
| Morgan et al., 1993 | TAU | BIC | In person | 32.5 | na | 0.0 | 23.0 | na | Regardless |
| Allard et al., 1992 | TAU | Psychodynamic | In person | na | na | 51.0 | na | 54.0 | With |
| Torhorst et al., 1988 | Psychodynamic | Psychodynamic | Mixed | na | na | 100.0 | na | na | With |
| Hawton et al., 1981 | PST | PST | In person | 25.3 | 9.0 | 67.0 | na | 73.0 | Regardless |
| Gibbons et al., 1978 | TAU | Case management | Mixed | na | na | na | na | na | Regardless |
| Welu, 1977 | TAU | BIC | Mixed | na | na | na | na | na | With |

Note:

*Armitage et al., 2016 tested Supportive therapy and Help Sheet vs TAU

**Wei et al., 2013 tested BIC and CBT interventions vs TAU

TAU = Treatment as usual.

“Regardless” = Regardless of suicidal intent; “With” = With suicidal intent.

Interventions definitions:

- Brief intervention and contact (BIC) might include some in person contact and may include letters, text messages, telephone calls, and postcards. It seeks to maintain contact with patients, providing a sense of ongoing concern, mitigating the sense of social isolation, as well as providing some psychoeducation, such as knowledge about triggers, warning signs for SH, and alternative coping strategies to SH. Source: Witt et al., 2021.
- Case management is an intervention which goal is to assess the patient's needs, developing a care plan, providing the means so it can be implemented and monitoring the results. It might have a significant role in increasing treatment adherence, as it includes one assigned professional to organize the case and because it stimulates continuous contact with the patient. Source: Witt et al., 2021
- Cognitive Behavioral Therapy (CBT) for suicide prevention focuses on helping high-risk individuals to identify specific factors that trigger and intensify suicidal thoughts and behaviors, as well as to develop effective coping strategies when dealing with stressors and problems that activate their suicidality. Source: Bryan & Rudd, 2018; Mann et al., 2021.
- Family-based interventions usually target young people at risk of suicide and can range from individual treatments encompassing a family component to treatments that focus specifically on the family. Source: Krysinska & Andriessen, 2022.
- Guideline for GP: this intervention focuses on educating non-psychiatrist physicians. The goal is to provide training to primary care health professional, usually including doctors and nurses, at the local and state level to better screen and assist patients with suicide related demands. Source: Mann et al., 2021.
- Problem-solving therapy (PST) is a CBT-based intervention that can be delivered as a therapy in and of itself, however, is also a component that integrates CBT therapies. The main treatment goal is to help patients to develop a problem-solving orientation, using rational and effective strategies, reducing the tendency to avoid problem-solving, and reduce the use of impulsive problem-solving strategies. Source: Witt et al., 2021
- Psychodynamic approaches for suicide prevention focus on understanding the unconscious meaning of SH, and exploring difficulties in interpersonal relationships, within a therapeutic relational framework. Usually psychodynamic therapies are not manualized, but here we have also incorporated Mentalization-based therapy (MBT) as a form of psychodynamic intervention. Source: Witt et al., 2021.
- Safety planning interventions are one type of brief intervention, which goal is to reduce the imminent risk of suicidal behavior by constructing a predetermined plan, containing a set of coping strategies and sources of support. Source: Nuji et al, 2021.
- Supportive Therapy contemplates interventions that rely on common factors only, focusing on building a strong therapeutic alliance, providing empathy, reassurance, and encouragement, and enhancing the client's strengths and coping mechanisms. It is a non-structured therapy, not focused on specific techniques derived by specific theoretical approaches. Source: Grover et al., 2020.
- Therapeutic assessments usually aim to screen for suicide risk to identify otherwise undetected at-risk individuals. Source: Mann et al., 2021.
- A help sheet intervention is a structured tool that facilitates the formation of implementation intentions by prompting individuals to link predefined high-risk situations (“if”) with adaptive coping responses (“then”). It aims to automatize protective behaviors in critical situations, enhancing self-regulation through strategic cue-response associations. Source: Armitage CJ., 2008.
- Selective serotonin reuptake inhibitors (SSRIs)

**Supplementary Material 4.** List of excluded studies.

| **Study** | **Design** | **Reason for exclusion** |
| --- | --- | --- |
| Westling et al., 2019 | RCT | Not all participants had an episode of self-harm within one month of their admission. The study also encompassed individuals exhibiting recurrent SB with a history of multiple admissions to emergency departments. |
| Domany et al., 2020 | RCT | Not all participants had presented with self-harm. The study included participants with SI. |
| Boege et al., 2022 | CT | Lack of randomization. |
| Luxton et al., 2014 | RCT | Not all participants had presented with self-harm. |
| Boudreaux et al., 2020 | Implementation Trial | Not a RCT. |
| Hughes and Asarnow, 2013 | RCT | Not all participants had presented with self-harm. The study included participants with SI. |
| Mehlum et al., 2014, 2016 | RCT | Not all participants had an episode of self-harm within one month of their admission. The inclusion criteria were a history of at least 2 episodes of self-harm, at least 1 within the last 16 weeks. |
| Shivanekar et al., 2022 | Pilot CT | Lack of randomization. |
| Cebrià et al., 2013 | CT | Lack of randomization. |
| Comtois et al., 2023 | RCT | Not all participants had an episode of self-harm within one month of their admission. The inclusion criterion was expanded from the past month to a lifetime history of SA, and acute hospitalizations aimed at preventing suicide were considered as part of events related to SB. |
| King et al., 2006, 2009 | RCT | Not all participants had had presented with self-harm. The study included participants with SI. |
| Esposito-Smythers et al., 2011 | RCT | Not all participants had had presented with self-harm. The study included participants with SI. |
| Patsiokas and Clum, 1985. | RCT | Does not specify admission timing post-self-harm event. |
| Gratz et al., 2014 | RCT | Not all participants had an episode of self-harm within one month of their admission. The inclusion criteria were a history of repeated self-harm, with at least one episode in the past six months of the admission. |
| George et al., 2014 | RCT | Not all participants had presented with self-harm.. The study included participants with SI. |
| Tolliver et al., 2016 | RCT | Not all participants had an episode of self-harm within one month of their admission. |
| Canuso et al., 2019, 2021 Ionescu et al., 2021 (ASPIRE Study) | RCT | Not all participants had presented with self-harm. The study included participants with active SI. |
| De Leo and Heller, 2007 | RCT | Not all participants had presented with self-harm. The study included participants with severe SI. |
| Jobes et al., 2007  Gutierrez et al., 2022 | RCT | Not all participants had presented with self-harm. The study included participants with SI. |
| Michaels et al., 2018 | RCT | Not all participants had presented with self-harm. The study included participants with SI and mood or alcohol abuse disorder. |
| Johnson et al., 2017 | RCT | Not all participants had presented with self-harm. The study included participants with SI. |
| Goodman et al., 2021 | Open trial | Not an RCT. |
| Asarnow et al., 2014 (SAFETY Program) | Treatment-development trial | Lack of randomization and inclusion criteria were SA within the past 3 months. |
| Huey et al., 2009 Henggeler et al., 1999, 2003 | RCT | Not all participants had presented with self-harm. The study included participants with SI. |
| Brent et al. 2009 (TASA study) | Open Treatment Trial | Lack of randomization and inclusion criteria were SA within the past 3 months. |
| Wharff et al., 2019 | RCT | Not all participants had presented with self-harm, and “suicidality” was self/parental referral. |
| Grupp-Phelan et al., 2012 | RCT | Not all participants had presented with self-harm. |
| Rogers et al., 2014 | Retrospective chart review | Not an RCT. |
| Ramsey et al., 2021 | RCT | Not all participants had an episode of self-harm within one month of their admission. The inclusion criteria were a history of at least 2 episodes of self-harm, at least 1 within the last 16 weeks. |
| Robinson et al., 2012 | RCT | Not all participants had presented with self-harm. The study included participants with SI. |
| Esposito-Smythers et al., 2019 | RCT | Does not specify admission timing post-self-harm event and includes participants with SI. |
| Czyz et al. 2021 | RCT | Not all participants had presented with self-harm. The study included participants with SI. |
| Sun et al., 2014 | RCT | Not all suicidal relatives of the caregivers experienced a self-harm episode/SA within one month prior to the caregiver's admission to the study. |
| Norrie et al., 2013 (BOSCOT Study) | RCT | Not all participants had an episode of self-harm within one month of their admission. The study included participants with a history of self-harm in the previous 12 months of their admission. |
| Gabilondo et al., 2020 | CT | Lack of randomization. |
| Luxton et al., 2020 | RCT | Not all participants had presented with self-harm. |
| Zullo et al., 2021 | Pilot CT | Lack of randomization. |
| Ghahramanlou-Holloway et al., 2012, 2020 Novak et al., 2023 (PACT study) | RCT | Not all participants had presented with self-harm. The study also included patients with SI with a history of a prior suicide attempt. |
| Vaiva et al., 2006 | RCT | Not all participants had an episode of self-harm within one month of their admission. Researchers contacted participants one month or three months after discharge from an emergency department for attempted suicide. |
| Prieb et al., 2012 Barnicot et al., 2014 | RCT | Not all participants had an episode of self-harm within one month of their admission. Participants had 5 or more episodes of self-harm in the year prior to the intervention. |
| Bateman et al., 2009 | RCT | Not all participants had an episode of self-harm within one month of their admission. The study included participants with a history of self-harm in the previous 6 months of their admission. |
| Oquendo et al., 2011 | RCT | Not all participants had an episode of self-harm within one month of their admission. The inclusion criteria were at least one past SA. |
| Rengasamy et al., 2019 | RCT | Not all participants had presented with self-harm. The study included participants with SI. |
| Riblet et al., 2021 | Pilot RCT | Not all participants had presented with self-harm. The study included patients admitted due to acute risk for self-harm. |
| Asarnow et al., 2011 | RCT | Not all participants had presented with self-harm. The study included participants with SI. |
| Hurtado-Santiago et al., 2022 | Preliminary RCT | Not all participants had presented with self-harm. The study included participants with SI. |
| Owens et al., 2020 | RCT | Not all participants had an episode of self-harm within one month of their admission. Participants had self-harm 6 weeks prior to the intervention. |
| Haddock et al., 2019 | RCT | Not all participants had presented with self-harm. The study included participants with SI. |
| Waterhouse et al., 1990 | RCT | Does not specify admission timing post parasuicidal act. |
| Winter et al., 2007 | Controlled Trial | Lack of randomization. |
| Rathus and Miller, 2002 | Controlled Trial | Lack of randomization. |
| van Landschoot et al., 2017 | RCT | Not all patients of the participant staff had presented with self-harm. |
| Goodman et al., 2016 | RCT | Not all participants had presented with self-harm. The study included participants with SI. |
| Interian et al., 2021 | RCT | Not all participants had presented with self-harm. The study included participants with SI. |
| Goñi-Sarriés et al., 2022 | Open Trial | Lack of randomization. |
| Hawton et al., 1987 | RCT | Inclusion criteria of subjects does not mention self-poisoning, but overdose. |
| Green et al., 2011 | RCT | Not all participants had an episode of self-harm within one month of their admission. The study included participants with a history of 2 or more presentations with self-harm in the previous 12 months of their admission. |
| Griffiths et al., 2019 | Pilot RCT | Not all participants had an episode of self-harm within one month of their admission. Participants had self-harm 6 months weeks prior to the intervention. |
| Primack et al., 2022 | RCT | Not all participants had an episode of self-harm. The study included participants with SI. |
| Linehan et al., 1991, 1993, 1994 | RCT | Not all participants had an episode of self-harm within one month of their admission. The study included patients who had at least two incidents of parasuicide in the last 5 years, with one during the last 8 weeks. |
| Cedereke et al, 2002 | RCT | Although the assessment was 49 days after the index SA, the intervention was 1 year after the SA. |
| Tormoen et al, 2014 | RCT | Not all participants had an episode of self-harm within one month of their admission. The inclusion criteria were more than one lifetime episode of self-harm with one of the episodes within the last  4 months before referral. |
| Green et al., 2011 | RCT | Not all participants had an episode of self-harm within one month of their admission. The inclusion criteria were at least two episodes of self-harm during the previous year. |
| Hazeli et al, 2009 | RCT | Not all participants had an episode of self-harm within one month of their admission. The inclusion criteria were at least two episodes of self-harm in the past year, one of which had occurred in the past 3 months. |
| O'Toole, 2019 | RCT | Not all participants had presented with self-harm. The study included participants with current SI with or without a history of suicide attempt, |
| Chen et al., 2012 | CT | Lack of randomization. |
| Bryan et al., 2018 | RCT | Not all participants had presented with self-harm. The study included participants with SI. |
| Rudd et al., 2015 | RCT | Not all participants had presented with self-harm. The study included participants with SI. |
| Kennard et al., 2018 | RCT | Not all participants had presented with self-harm. The study included participants with SI. |
| Raj et al., 2011 | Quasi-experimental | Quasi-experimental study. Participants were allocated sequentially to experimental and control groups. |
| Sedghy et al., 2020 | Quasi-experimental | Quasi-experimental study. Participants were included through  convenience sampling and the randomization method was controversial. |
| Harned et al., 2014 | RCT | According to the inclusion criteria, participants may have engaged in self-harm in the past 8 weeks (> 1 month). |
| Chowdhury, Hicks & Kreitman, 1973 | CT | Lack of randomization. |
| Kleiman et al., 2024 | RCT | Also included participants with admission due to severe suicidal thinking. |
| Zhang et al., 2024 | RCT | Paper only available in Chinese. Reviewers weren't able to verify inclusion criteria and extract data. |
| Stallard et al., 2024 | RCT | Not all participants had an episode of self-harm within one month to their admission, although 91% reported self-harm in the past <30 days. |
| Marasinghe et al., 2012 | RCT | The study does not justify why the outcome of new self-harm was not reported. |
| Spirito et al., 2002 | RCT | The outcome new self-harm was not reported |
| Rossouw et al., 2012 | RCT | Reviewers were unable to understand how the authors reported new self-harm and could not contact them for clarification. |
| Diefenbach et al., 2024 | RCT | Not all participants had engaged in self-harm within one month prior to admission; the study also included individuals who had attempted suicide within the past two years, provided they were currently experiencing suicidal ideation with a plan. |
| Goldstein et al., 2025 | RCT | Included participants who were hospitalized for suicidal ideation with plan and/or intent. |
| Keyworth et al., 2025 | RCT | Not all participants had engaged in self-harm within one month prior to admission. |
| Monn et al., 2025 | RCT | Not all participants had engaged in self-harm within one month prior to admission. The protocol was modified compared to the 2016 trial to include participants with a suicide attempt within the previous 6 months. |

*Note.*

*Papers unavailable in any resources for full text analysis:*

*Draper R, Hirsch S. Treatment of parasuicide patients with mianserin, nomifensine and placebo, a double-blind placebo-controlled trial. Unpublished 1982.*

*Quinn M, Bozzay ML, McGeary J, Primack JM. Determining suicide risk in high-risk veterans hospitalized for suicidality: the predictive utility of alcohol use. Alcoholism: Clinical and Experimental Research Volume 46: 45th Annual Speaker & Poster Abstracts of the Research Society on Alcoholism, June 2022.*

*Zhang Chunmiao, Li Xianyun, Xu Ruoyu, et al. A randomized controlled study on the efficacy of cognitive behavioral therapy for suicide prevention in patients with depression who attempted suicide[J]. Chinese Journal of Psychiatry, 2024, 57(09): 570-578.*

Abbreviation Key: RCT = Randomized Controlled Trial; SA = Suicide attempt; SI = Suicide ideation; SB = Suicide behavior

**Supplementary Material 5.** Characteristics of randomized controlled trials included in our review

|  | **k (%) / mean (SD) / median (IQR)** |
| --- | --- |
| Recruiting area – number of studies (%) | UK: 14 (23.3%); USA: 7 (11.7%); Iran: 4 (6.7%); New Zealand: 4 (6.7%); Others: 31 (51.7%) |
| **Year – mean (SD)** |  |
| Study started | 2004.0 (11.2) |
| Study completed recruitment | 2005.8 (11.3) |
| Study published | 2009.0 (11.4) |
| **Funding – number of studies (%)** |  |
| Non-industry | 53 (88.3%) |
| At least partially industry | 7 (11.7%) |
| **Multicenter status – number of studies (%)** |  |
| Multicenter | 16 (26.7%) |
| Single center | 44 (73.3%) |
| RCT masking – number of studies (%) | Assessor-masked: 30 (50.0%); Double-blinded: 3 (5.0%); Single-blinded: 1 (1.7%); Unblinded: 17 (28.3%); Unclear: 9 (15.0%) |
| Analysis – number of studies (%) | ITT: 42 (70.0%); Zelen: 5 (8.3%); Completers (modified ITT): 12 (20.0%); Unclear: 1 (1.7%) |
| Number of participants randomized – total; median (IQR) | 22,654; 185 (88–449) |
| **Age group** |  |
| Only children and adolescents – number of studies (%) | 4 (6.7%) |
| Only adolescents – number of studies (%) | 2 (3.3%) |
| Adolescents and adults – number of studies (%) | 20 (33.3%) |
| Only adults – number of studies (%) | 32 (53.3%) |
| Missing (%) | 2 (3.3%) |
| Mean age – mean (SD) | 31.8 (6.3) |
| Age – median (IQR) | 32 (27–33) |
| Not married – mean (% missing) | 61.4% (43.3% missing) |
| Female sex – mean (% missing) | 66.1% (15.0% missing) |
| **Race** |  |
| Black – mean (%) | 14.3% |
| White – mean (%) | 65.5% |
| Asian – mean (%) | 5.4% |
| Hispanic – mean (%) | 7.0% |
| Missing race information | 93.3% missing in at least one race variable |
| **Suicidal intent requirement – number of studies (%)** |  |
| With suicidal intent | 21 (35.0%) |
| Regardless of suicidal intent | 39 (65.0%) |
| Repeat self-harm (“repeaters”) – mean (% missing) | 55.9% (21.7% missing) |
| **Diagnostic – mean (% missing)** |  |
| MDD | 57.5% (80.0% missing) |
| BPD | 66.9% (95.0% missing) |
| **Intervention** |  |
| Number of sessions – mean (range) | 7.5 (0.0–58.0) |
| Duration of follow-up – mean (SD) | 15.1 (12.7) |
| Format (by study arms, n = 123) – number of studies (%) | Individual: 74 (60.2%); Group/Mixed: 4 (3.2%); Missing 45 (36.6%) |
| Delivery (by study arms, n = 123) – number of studies (%) | In person: 80 (65%); Mixed: 16 (13%); Remote: 10 (8.1%); Missing: (13.8%) |

**Supplementary Material 6.** RoB 2 Summary by Study and Domain

**Supplementary Material 7.** Publication bias: funnel plots with trim-and-fill.

Legend: Gray circles indicate included studies (numbered: 1, Brown et al., 2005; 2, Conner et al., 2021; 3, Evans et al., 2005; 4, Gysin-Maillart et al., 2016; 5, Hatcher et al., 2015; 6, Hatcher et al., 2016; 7, Husain et al., 2014; 8, LaCroix et al., 2018; 9, Mousavi et al., 2014; 10, Mousavi, Tehrani & Maracy, 2017; 11, R. C. O'Connor et al., 2017; 12, Stevens et al., 2024; 13, Tyrer et al., 2003; 14, Vaiva et al., 2018; 15, Van Der Sande et al., 1997; 16, Wei et al., 2013; 17, Wei et al., 2013, 18, Welu, 1977), while white circles represent studies imputed by the trim-and-fill procedure. Wei et al., 2013 represented twice because two interventions (BIC and CBT) were compared with TAU (control) and, therefore, were analysed separately.

Legend: Gray circles indicate included studies (numbered: 1, Beautrais et al., 2010; 2, Brown et al., 2005; 3, Carter et al., 2013; 4, Clarke et al., 2002; 5, Cotgrove et al., 1995; 6, Evans et al., 2005; 7, Gibbons et al., 1978; 8, Gysin-Maillart et al., 2016; 9, Hassanian-Moghaddam et al., 2017; 10, Hatcher et al., 2011; 11, Hatcher et al., 2015; 12, Hatcher et al., 2016; 13, Kapur et al., 2013; 14, Malakouti et al., 2022; 15, McAuliffe et al., 2014; 16, Morgan et al., 1993; 17, Morthorst et al., 2012; 18, Mouaffak et al., 2015; 19, Mousavi, Tehrani & Maracy, 2017; 20, Stevens et al., 2024; 21, Tyrer et al., 2003; 22 Van Der Sande et al., 1997; 23, Van Heeringen et al., 1995; 24, Wei et al., 2013; 25, Wei et al., 2013), while white circles represent studies imputed by the trim-and-fill procedure. Wei et al., 2013 represented twice because two interventions (BIC and CBT) were compared with TAU (control) and, therefore, were analysed separately.

**Supplementary Material 8.** New Suicide Deaths at each time-point, including double-zero studies.

**
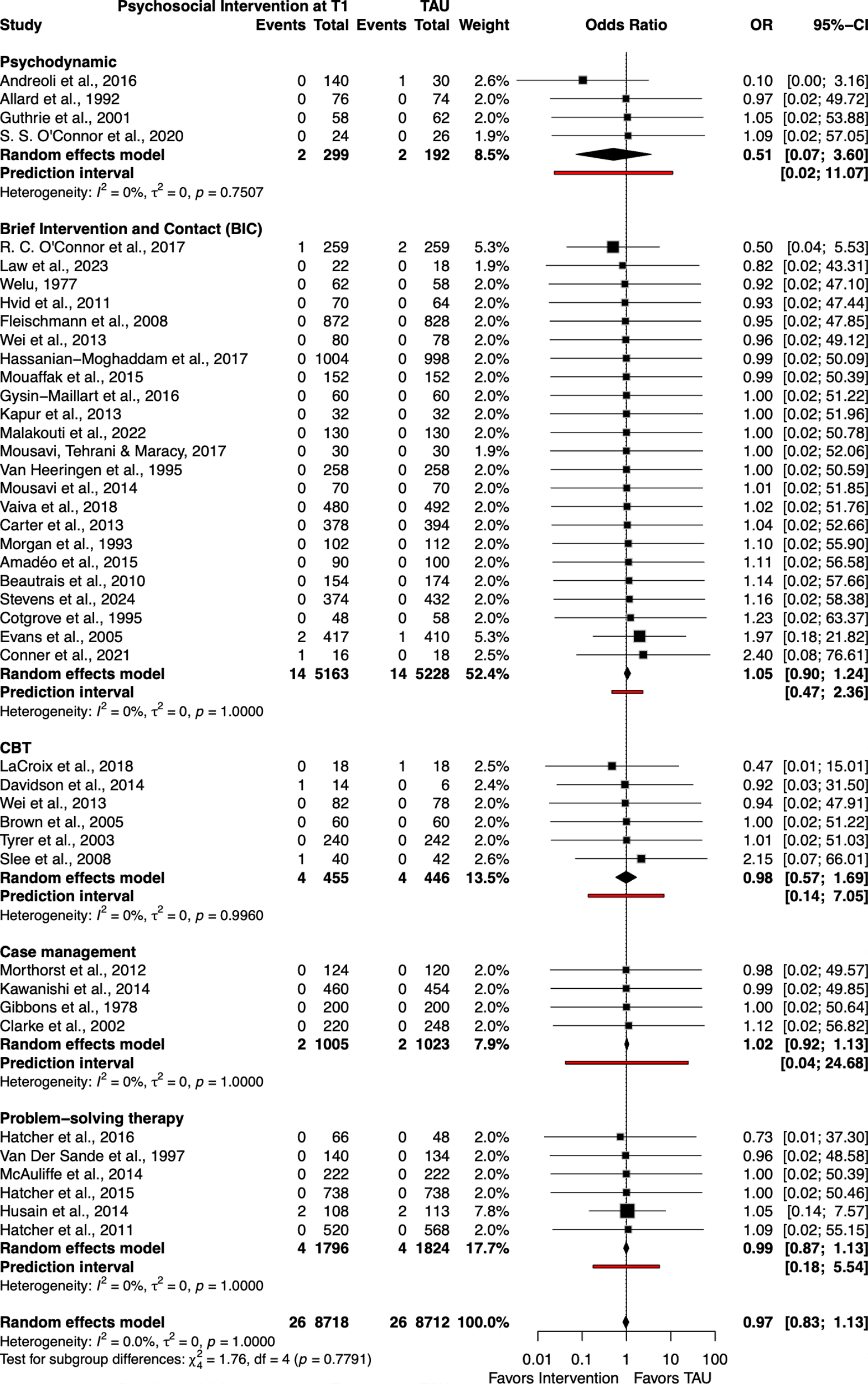
**

**
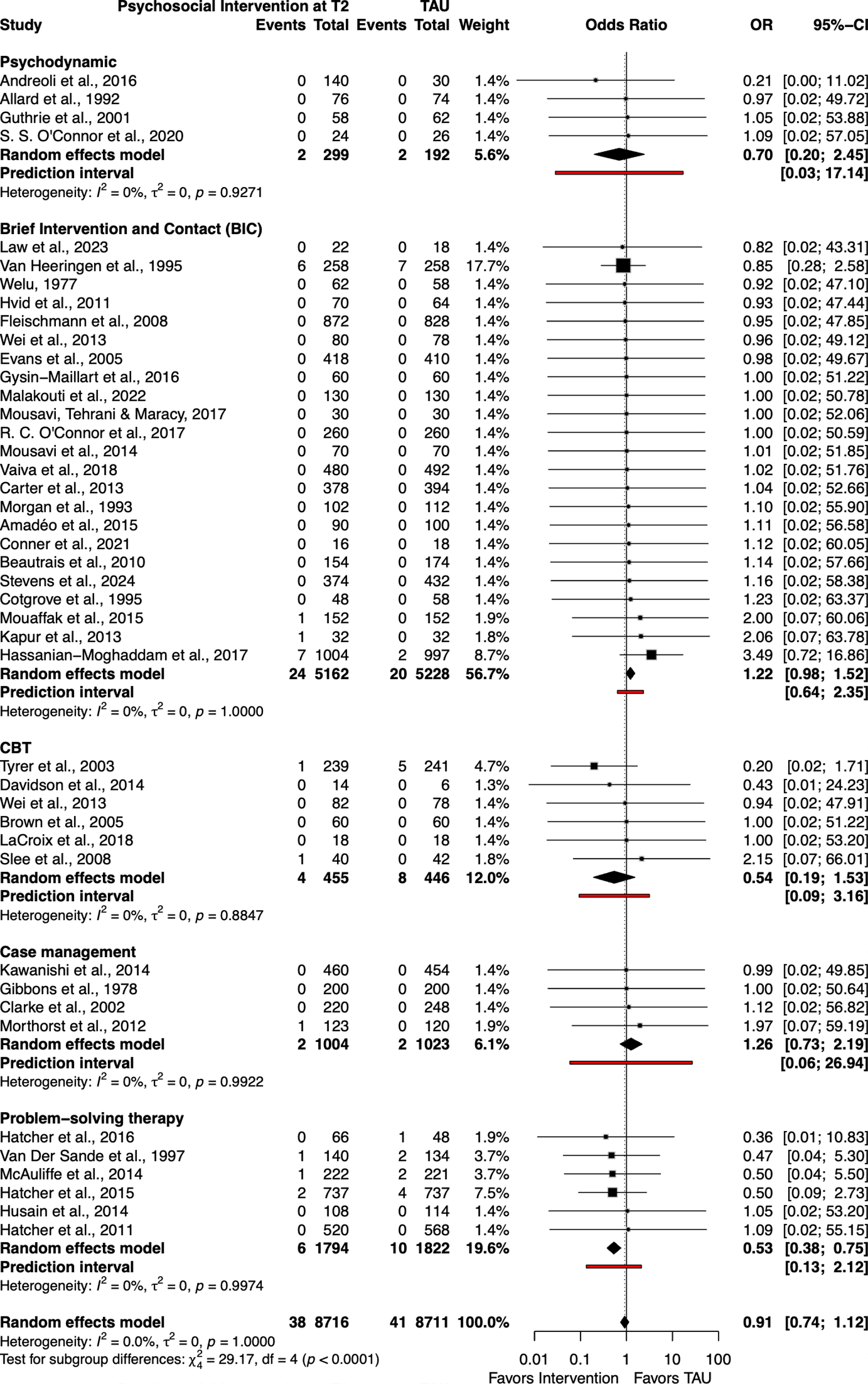
**

**
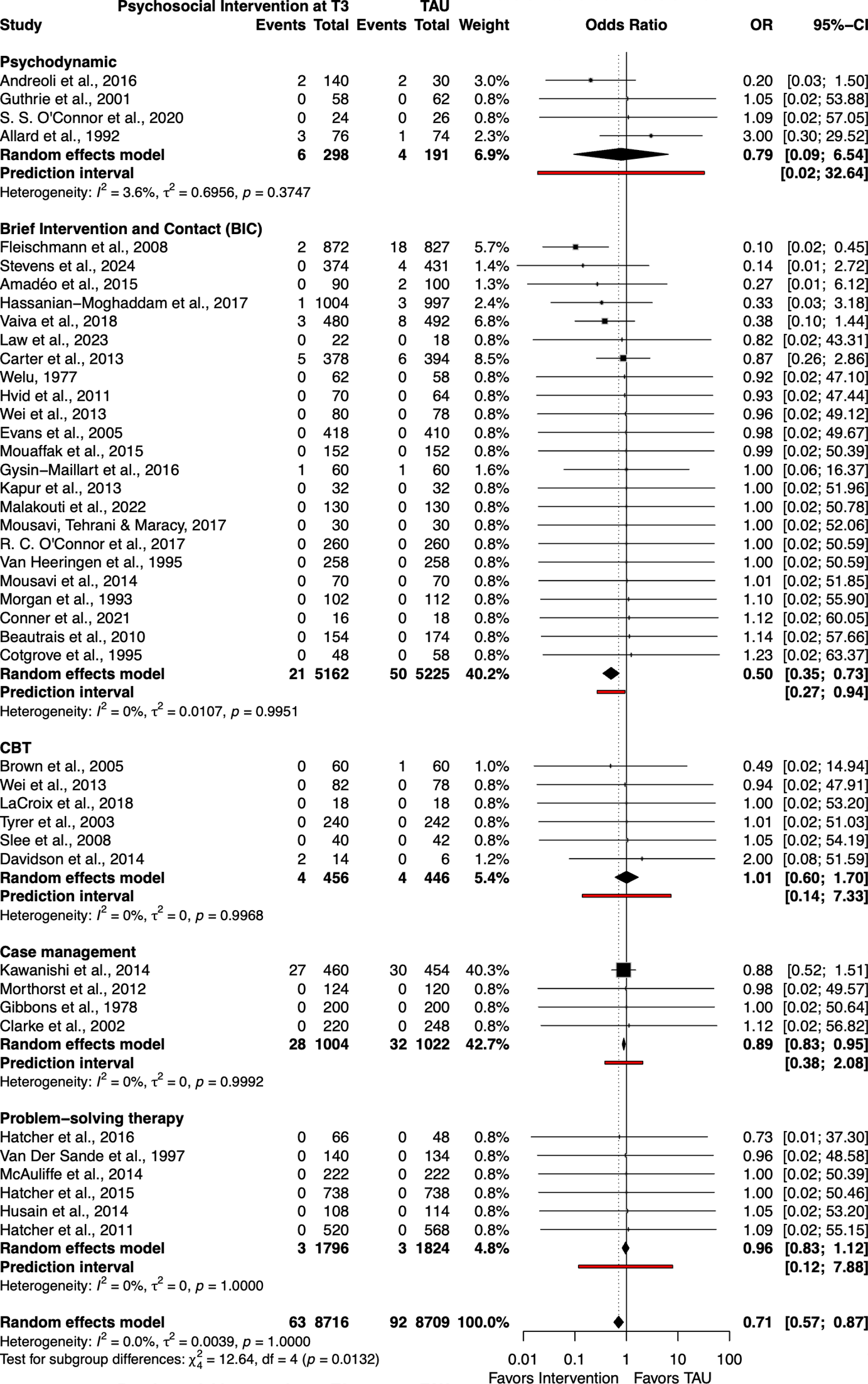
**

**Supplementary Material 9.** Psychosocial Interventions vs. TAU: New Self-Harm at T2 by Suicidal Intent (Regardless vs With suicidal intent).

**Supplementary Material 10.** Bubble plots for suicidal intent and mean age at T2.

**Supplementary Material 11.** GRADE evidence tables.

**Question:** CBT compared to TAU for preventing self-harm recurrence.

**Setting:** Individuals with recent self-harm (≤1 month), hospital/clinical settings.

| **Certainty assessment** | | | | | | | **№ of patients** | | **Effect** | | **Certainty** | **Importance** |
| --- | --- | --- | --- | --- | --- | --- | --- | --- | --- | --- | --- | --- |
| **№ of studies** | **Study design** | **Risk of bias** | **Inconsistency** | **Indirectness** | **Imprecision** | **Other considerations** | **CBT** | **TAU** | **Relative (95% CI)** | **Absolute (95% CI)** |  |  |
| **Self-harm at T1 (follow-up: range 0 to 6 months)** | | | | | | | | | | | | |
| 4 | randomised trials | serious^a^ | not serious | not serious | very serious^b^ | none | 73/316 (23.1%) | 99/327 (30.3%) | **OR 0.57** (0.17 to 1.87) | **104 fewer per 1,000** (from 234 fewer to 145 more) | ⨁◯◯◯ Very low^a,b^ | CRITICAL |
| **Self-harm at T2 (follow-up: range >6 months to 12 months)** | | | | | | | | | | | | |
| 3 | randomised trials | serious^c^ | not serious | not serious | very serious^d^ | publication bias strongly suspected^e^ | 97/287 (33.8%) | 125/293 (42.7%) | **OR 0.60** (0.17 to 2.03) | **118 fewer per 1,000** (from 314 fewer to 175 more) | ⨁◯◯◯ Very low^c,d,e^ | CRITICAL |

Legend:

CI: confidence interval; OR: odds ratio

a. Two studies at low risk of bias, one with some concerns, and one at high risk; downgraded one level for risk of bias.

b. Wide 95% CI (0.17–1.87) spans substantial benefit, no effect, and potential harm; total events = 172 (<300). Downgraded two levels for imprecision.

c. Include two studies at low risk of bias and one at high risk. Downgraded one level for risk of bias.

d. Very wide 95% CI (0.17–2.03) spanning substantial benefit, no effect, and potential harm; total events = 222 (<300). Downgraded two levels for imprecision.

e. Mild funnel plot asymmetry, significant Egger’s test (p = 0.0152), and trim-and-fill imputed 4 studies; publication bias strongly suspected.

**Question:** BIC compared to TAU for preventing self-harm recurrence.

**Setting:** Individuals with recent self-harm (≤1 month), hospital/clinical settings.

| **Certainty assessment** | | | | | | | **№ of patients** | | **Effect** | | **Certainty** | **Importance** |
| --- | --- | --- | --- | --- | --- | --- | --- | --- | --- | --- | --- | --- |
| **№ of studies** | **Study design** | **Risk of bias** | **Inconsistency** | **Indirectness** | **Imprecision** | **Other considerations** | **BIC** | **TAU** | **Relative (95% CI)** | **Absolute (95% CI)** |  |  |
| **Self-harm at T1 (follow-up: range 0 to 6 months)** | | | | | | | | | | | | |
| 10 | randomised trials | serious^a^ | not serious | not serious | serious^b^ | none | 266/1762 (15.1%) | 299/1810 (16.5%) | **OR 0.88** (0.62 to 1.26) | **17 fewer per 1,000** (from 56 fewer to 34 more) | ⨁⨁◯◯ Low^a,b^ | CRITICAL |
| **Self-harm at T2 (follow-up: range 6 months to 12 months)** | | | | | | | | | | | | |
| 14 | randomised trials | serious^a^ | serious^c^ | not serious | not serious | none | 384/3138 (12.2%) | 469/3264 (14.4%) | **OR 0.75** (0.52 to 1.08) | **32 fewer per 1,000** (from 63 fewer to 10 more) | ⨁⨁◯◯ Low^a,c^ | CRITICAL |
| **Self-harm at T3 (follow-up: range 12 months to)** | | | | | | | | | | | | |
| 7 | randomised trials | serious^a^ | serious^d^ | not serious | not serious | none | 403/2385 (16.9%) | 516/2431 (21.2%) | **OR 0.77** (0.53 to 1.12) | **40 fewer per 1,000** (from 87 fewer to 20 more) | ⨁⨁◯◯ Low^a,d^ | CRITICAL |

Legend:

CI: confidence interval; OR: odds ratio

a. Several studies at high risk of bias (e.g., Wei 2013, Stevens 2024...), others with some concerns; downgraded one level for risk of bias.

b. 95% CI (0.62–1.26) crosses the null and spans appreciable benefit and harm; total events = 565 (>300). Downgraded one level for imprecision.

c. Substantial heterogeneity (I² = 62%); effect sizes varied from large benefit to possible harm. Downgraded one level for inconsistency.

d. Substantial heterogeneity (I² = 62%); effect estimates ranged from large benefit (Gysin 2016) to possible harm (Amadéo 2015). Downgraded one level for inconsistency.

**Question:** PST compared to TAU for preventing self-harm recurrence.

**Setting:** Individuals with recent self-harm (≤1 month), hospital/clinical settings.

| **Certainty assessment** | | | | | | | **№ of patients** | | **Effect** | | **Certainty** | **Importance** |
| --- | --- | --- | --- | --- | --- | --- | --- | --- | --- | --- | --- | --- |
| **№ of studies** | **Study design** | **Risk of bias** | **Inconsistency** | **Indirectness** | **Imprecision** | **Other considerations** | **PST** | **TAU** | **Relative (95% CI)** | **Absolute (95% CI)** |  |  |
| **Self-harm at T1 (follow-up: range 0 to 6 months)** | | | | | | | | | | | | |
| 4 | randomised trials | not serious | not serious | not serious | very serious^a^ | none | 122/670 (18.2%) | 108/676 (16.0%) | **OR 1.18** (0.78 to 1.79) | **23 more per 1,000** (from 31 fewer to 94 more) | ⨁⨁◯◯ Low^a^ | CRITICAL |
| **Self-harm at T2 (follow-up: range 6 months to 12 months)** | | | | | | | | | | | | |
| 5 | randomised trials | serious^b^ | not serious | not serious | serious^c^ | none | 324/1306 (24.8%) | 315/1346 (23.4%) | **OR 1.07** (0.87 to 1.32) | **12 more per 1,000** (from 24 fewer to 53 more) | ⨁⨁◯◯ Low^b,c^ | CRITICAL |

Legend:

CI: confidence interval; OR: odds ratio

a. 95% CI (0.78–1.79) crosses the null, ranging from moderate benefit to harm; total events = 230 (<300). Downgraded two levels for imprecision.

b. Three trials at low risk (Hatcher 2016; Hatcher 2011; Van der Sande 1997), one with some concerns (Hatcher 2015), and one at high risk (McAuliffe 2014); downgraded one level for risk of bias.

c. 95% CI (0.87–1.32) crosses the null, ranging from no effect to possible harm; total events = 639 (>300). Downgraded one level for imprecision.

**Question:** Case Management compared to TAU for preventing self-harm recurrence

**Setting:** Individuals with recent self-harm (≤1 month), hospital/clinical settings.

| **Certainty assessment** | | | | | | | **№ of patients** | | **Effect** | | **Certainty** | **Importance** |
| --- | --- | --- | --- | --- | --- | --- | --- | --- | --- | --- | --- | --- |
| **№ of studies** | **Study design** | **Risk of bias** | **Inconsistency** | **Indirectness** | **Imprecision** | **Other considerations** | **Case Management** | **TAU** | **Relative (95% CI)** | **Absolute (95% CI)** |  |  |
| **Self-harm at T1 (follow-up: range 0 to 6 months)** | | | | | | | | | | | | |
| 3 | randomised trials | not serious | not serious | not serious | very serious^a^ | none | 66/543 (12.2%) | 67/567 (11.8%) | **OR 1.02** (0.47 to 2.22) | **2 more per 1,000** (from 59 fewer to 111 more) | ⨁⨁◯◯ Low^a^ | CRITICAL |

Legend:

CI: confidence interval; OR: odds ratio

a. Wide 95% CI (0.47–2.22) spanning substantial benefit, no effect, and harm; total events = 133 (<300). Downgraded two levels for imprecision.
